# AlphaFold‐driven discovery of oxysterol‐binding protein‐related protein‐phosphoinositide 3‐, 4‐, and 5‐phosphatase interactions using new generation confidence scores

**DOI:** 10.1002/pro.70572

**Published:** 2026-04-15

**Authors:** Filippo Dall'Armellina, Sylvie Urbé, Daniel J. Rigden

**Affiliations:** ^1^ Department of Biochemistry, Cell and Systems Biology Institute of Systems, Molecular and Integrative Biology, University of Liverpool Liverpool UK

**Keywords:** AlphaFold structural prediction, membrane contact sites, non‐vesicular lipid transport, ORPs, SAC1

## Abstract

Non‐vesicular lipid transport contributes to the regulation of membrane composition and organelle function at membrane contact sites. OSBP‐related proteins (ORPs) are central to this process, yet their interaction networks remain incompletely defined. Here, we systematically screened potential interactions between ORPs and phosphoinositide 3‐, 4‐, and 5‐phosphatases using AlphaPulldown2, AlphaFold2‐Multimer, and AlphaFold3. We established a protocol for model generation by combining AlphaFold2‐Multimer predictions (including five‐replicates) with an AlphaPulldown2 interaction screen across around 200 protein pairs, and with AlphaFold3 predictions including lipid‐bound and multimeric assemblies. Interface confidence was assessed for consistency using the weighted ipTM + pTM metric, actifpTM, new generation ipSAE scoring, and FoldSeek‐Multimer clustering. We further evaluated the protein pairs' biological plausibility based on subcellular localization data, in silico membrane insertion, evolutionary conservation via ConSurf, and protein binding interface analysis using the deep learning tool PeSTo. This integrative protocol uncovered functionally conserved binding modes in the SAC1 lipid phosphatase with the ORP family, particularly with ORP11, and predicted functionally relevant protein‐lipid interfaces.

## 1 INTRODUCTION

Membrane contact sites (MCSs) are specialized subcellular compartments where organelles are closely apposed, typically 10–40 nm apart, enabling non‐vesicular exchange of lipids, ions, and metabolites (Prinz et al., 2020; Voeltz et al., 2024). Transfer between membranes can occur through protein‐mediated mechanisms (shuttle or bridge models), vesicular transport, or, when membranes approach within 2–3 nm, direct lipid diffusion (Prinz et al., 2020; Saheki & De Camilli, 2017). Cargo includes ions, metabolites, and lipids. Among the lipid cargo, phosphoinositides (PIs) are particularly important. These phospholipids act both as precursors for second messengers and as organizers of protein complexes on organelle membranes (Di Paolo & De Camilli, 2006; Posor et al., 2022). PIs regulate processes including membrane architecture, cytoskeletal dynamics, and signal transduction (Balla, 2013; Balla et al., 2020; Di Paolo & De Camilli, 2006). Their functions depend on both localization and phosphorylation state. Kinases and phosphatases act at positions 3, 4, and 5 of the inositol ring to generate seven phosphorylated species (Davies et al., 2025).

Phosphatases are widely distributed across organelles, but several function at the endoplasmic reticulum (ER) or plasma membrane (PM), where many MCSs form. Well‐studied examples include: (1) OCRL, a 5‐phosphatase that functions within the secretory pathway, localizing to the trans‐Golgi network (TGN) and, under certain signals, to the PM (Faucherre et al., 2003); (2) INPP5K, another 5‐phosphatase, recruited to the ER surface by ARL6IP1 to hydrolyse PI(4,5)P2 (Dong et al., 2018) and also detected at the PM in signaling contexts (Yang et al., 2015); and (3) SAC1, an ER‐embedded 4‐phosphatase that hydrolyses PI4P molecules (Zewe et al., 2018). Together, these enzymes illustrate how phosphatases not only remove phosphate groups but also actively shape the lipid environment, preparing lipids for transfer at MCSs (Saheki & De Camilli, 2017).

The ER‐PM interface is a key MCS hosting many lipid transfer proteins (Saheki & De Camilli, 2017), including the oxysterol‐binding protein (OSBP)‐related proteins (ORPs) which mediate non‐vesicular lipid exchange between organelles (Cabukusta et al., 2024; Kawasaki et al., 2022; Nakatsu & Kawasaki, 2021; Olkkonen & Li, 2013; Zhou et al., 2010). ORPs are typically recruited to MCSs through interactions with VAMP‐associated proteins (VAPs) at the ER or with phosphatidylinositol 4‐phosphate (PI4P)‐enriched membranes (Olkkonen & Li, 2013), such as the PM or Golgi apparatus. Most ORPs contain a canonical FFAT motif (two phenylalanines in an acidic tract) that binds to the major sperm protein domain of VAPA/B transmembrane (TM) proteins embedded in the ER (Du et al., 2011; Kawasaki et al., 2022; Murphy & Levine, 2016). Beyond this, ORPs share three main domains: (1) a coiled‐coil domain, enabling dimerisation (e.g., ORP9‐ORP11) (Cabukusta et al., 2024; Zhou et al., 2010); (2) a pleckstrin homology (PH) domain; and (3) an OSBP‐related lipid‐binding (ORD) domain, accommodating ligands including sterols and PIs (Nakatsu & Kawasaki, 2021; Sohn et al., 2018).

ORPs can exchange cholesterol derivatives for other lipids across membranes and often functionally cooperate with SAC1, which dephosphorylates PI4P at ER‐PM contact sites (Del Bel & Brill, 2018). Together, ORPs and SAC1 generate and maintain lipid gradients across intracellular compartments (Del Bel & Brill, 2018; Moser von Filseck et al., 2015). SAC1 was initially proposed to act in trans (Manford et al., 2010), but subsequent studies suggest it acts in cis instead (Schafer et al., 2023; Stefan et al., 2011). In cis means that SAC1 dephosphorylates PI4P after the lipid has been transferred into the same membrane SAC1 is embedded in, the ER. Thus, PI4P is first extracted from the PM by an ORP, transferred to the ER, and then dephosphorylated by SAC1 within the ER membrane. In trans, SAC1 would act directly on PI4P located in the opposing membrane, the PM, without the lipid being transferred first. This requires SAC1's catalytic site to reach the adjacent membrane at an ER‐PM contact site. Given that SAC1 is an ER‐resident membrane protein, the “cis”‐model is structurally and biologically more plausible (Manford et al., 2010; Schafer et al., 2023).

It is worth noting that yeast expresses a different set of ORPs and Sac1 paralogs than humans. While prior studies in yeast suggested functional coupling between Osh proteins and Sac1, these experiments did not demonstrate a stable ORP‐SAC1 complex in solution. Crosslinking assays indicated proximity in vivo and were consistent with membrane‐dependent interactions. Furthermore, truncated Sac1 constructs in liposome assays were used to probe PI4P turnover rather than structural complex formation (Stefan et al., 2011). In contrast, our study models the interaction between human ORPs and SAC1, along with other PI phosphatase proteins. This approach provides structural and mechanistic insight into ORP‐SAC1 recognition and lipid transfer that was not accessible experimentally, complementing previous functional observations.

Given the central role of PI metabolism at MCSs, we hypothesized that transient or cooperative interactions between PI 3‐, 4‐, and 5‐phosphatases (PIPs) and lipid transfer proteins may facilitate substrate handoff or spatial regulation during cellular signaling events. We designed a modeling protocol with two state‐of‐the‐art structural prediction tools: AlphaPulldown2 and AlphaFold3. AlphaPulldown2, built on AlphaFold2‐Multimer, offers a scalable framework for all‐against‐all interaction screening (Molodenskiy et al., 2025). We treat AlphaFold2‐Multimer and AlphaFold3 as completely separate tools. AlphaFold2‐Multimer was used because it integrates scoring metrics beyond the default ipTM score: ipSAE and actifpTM (Dunbrack, 2025; Varga et al., 2025). By integrating outputs from both in silico methods and evaluating models using multiple confidence metrics (pLDDT, PAE, ipSAE, actifpTM), we mapped a landscape of potential interactions and contextualized them with subcellular localization datasets.

## 2 METHODS

### 2.1 Installation and use of LocalColabFold

LocalColabFold provides an installer script that enables ColabFold functionality on local machines, facilitating easy protein structure and complex prediction using AlphaFold2 and AlphaFold2‐Multimer. Multiple sequence alignments (MSAs) and template searches were generated using MMseqs2 and HHsearch (Jumper et al., 2021; Mirdita et al., 2022) and LocalColabFold used the UniRef30 database dated February 2023 (Evans et al., 2021; Jumper et al., 2021). LocalColabFold was installed via its GitHub repository (accessed April 2024, available at https://github.com/YoshitakaMo/localcolabfold) on a machine running Ubuntu 24.04.1 LTS (Noble) with kernel version 6.8.0‐51‐generic. The system was equipped with an AMD Ryzen Threadripper 3970X 32‐Core Processor (32 physical cores, 64 threads, 64 CPUs available) and supported both 32‐bit and 64‐bit operations. The machine had 125 GiB of RAM. For GPU computations, an NVIDIA GeForce RTX 3080 with 10 GB VRAM was used. The GPU was managed by NVIDIA‐SMI 535.183.01, running CUDA 12.2. During runtime, the GPU utilization remained low, and approximately 588 MiB of VRAM was in use, primarily by system processes. The software was executed in a Conda environment with Python 3.9 and necessary dependencies installed.

AlphaFold2 outputs several diagnostic plots and confidence metrics to assess the quality of its structural predictions. One such plot displays the coverage and depth of the initial MSA, alongside a map of regions covered in the full‐length protein. Among the key confidence scores is the predicted local distance difference test (pLDDT), which provides a per‐residue estimate of model accuracy on a scale from 0 to 100. Scores above 90 indicate very high local accuracy, comparable to experimentally determined structures, allowing reliable interpretation of side‐chain positioning. Scores between 70 and 90 reflect confident backbone predictions, while values between 50 and 70 suggest moderate confidence, where overall secondary structure elements (e.g., α‐helices and β‐strands) are likely correct, but their relative arrangement may be uncertain.

In addition to pLDDT, AlphaFold2 generates a predicted aligned error (PAE) plot, which estimates the positional error (in Angstroms) between all residue pairs in the model. The PAE is visualized as a 2D heatmap, where lower values indicate high confidence in the spatial relationship between residues. Typically, PAE values are low in well‐folded domains and higher between domains that may be flexibly linked or lack defined contact, reflecting uncertainty in their relative orientation. This makes PAE particularly useful for identifying flexible linkers or domain‐domain arrangements that may be functionally important but structurally dynamic.

### 2.2 AlphaFold3 and AlphaBridge web servers

The open access AlphaFold3 web server (accessed June 2024, available at https://alphafoldserver.com) was used to model single and complex protein structures, with and without ligands (Abramson et al., 2024). Protein sequences were submitted to the server in FASTA format. AlphaFold3 outputs were then uploaded to the publicly accessible Google Colab AF3_Results_Visualization.ipynb script (accessed February 2025, available at https://colab.research.google.com/github/Ash100/Biopython/blob/main/AF3_Results_Visualization.ipynb#scrollTo=YZAVOqx6dig5) to generate publication‐quality visualizations of inter‐residue error and confidence.

AlphaFold3 structural models were examined using the AlphaFold3 Visualiser script (Ahmad, n.d.) to inspect residue‐level interactions. For multimeric predictions, we further analyzed inter‐chain contacts using PDBePISA (Krissinel, 2010; Krissinel & Henrick, 2007). All models were visualized and interpreted in PyMOL (version 2.1.1, Schrödinger, LLC, New York, NY, USA), which was used for figure generation and manual inspection of predicted interfaces. The ORP11‐SAC1 complex was further analyzed for its interface residue data using AlphaBridge (Álvarez‐Salmoral et al., 2024). AlphaBridge is a tool that identifies and characterizes residue‐residue interactions in protein complex models generated by AlphaFold.

### 2.3 Analysis of AlphaPulldown2 and FoldSeek‐multimer cluster results

AlphaPulldown (v2) was downloaded from its GitHub repository (accessed September 2024, available at https://github.com/KosinskiLab/AlphaPulldown) to perform all‐against‐all structural interaction screens for multiple protein families (Molodenskiy et al., 2025; Yu et al., 2023). The input features needed for the protein modeling dataset were retrieved from the AlphaPulldown input features database (available at https://alphapulldown.s3.embl.de/input_features/index.html), which provides precomputed AlphaFold2 feature files (.pkl.xz) and accompanying metadata for 20,738 UniProt entries (Molodenskiy et al., 2025). These features were directly used as inputs for AlphaPulldown v1/v2 predictions. The number of recycles (−num‐recycles) was left at the default of 3, the model preset (monomer vs. multimer) was automatically determined, no templates were used or provided, and default MSA settings (−max‐seq) and random seed (−random‐seed) were applied.

To systematically evaluate potential protein–protein interactions at scale, we employed the AlphaFold3 server and AlphaPulldown2, a high‐throughput wrapper for AlphaFold‐Multimer designed for all‐against‐all structural interaction screens. AlphaPulldown2 leverages the underlying AlphaFold‐Multimer engine for structure prediction, and both AlphaPulldown2 and LocalColabFold utilize MMseqs2 for MSA generation. All predictions were generated using the same AlphaFold‐Multimer weights and inference code, ensuring no algorithmic differences at the neural network level between the tools. Procedural differences were limited to: (i) the use of distinct random seeds, which can introduce minor stochastic variation during recycling without altering the underlying model; (ii) differing ranking metrics (ipTM in LocalColabFold vs. actifpTM in AlphaPulldown2); and (iii) feature handling, with AlphaPulldown2 relying on precomputed MSA and template feature files from its input feature database (*Homo sapiens*), while AlphaFold2‐Multimer/LocalColabFold generated MSAs locally and this was done to obtain actifpTM values for the same protein pairs. Although both approaches ultimately drew from the same underlying sequence databases, the precomputed features in AlphaPulldown2 were generated by the developers between May and July 2025, whereas the LocalColabFold MSAs were generated in April 2024.

The resulting models from AlphaPulldown2 and AlphaFold3 were evaluated using multiple confidence metrics, including weighted ipTM + pTM, pDockQ, and ipSAE. These scores were used as primary filtering criteria to remove low‐confidence predictions. Only models exceeding all predefined confidence thresholds were retained for further analysis. AlphaFold‐Multimer was applied at a later stage using a local ColabFold implementation, and only to the subset of top‐ranking models that passed the initial confidence filtering. The actifpTM score was not used as a primary filtering criterion during the initial screening, as it is not available from AlphaPulldown2 or AlphaFold3 outputs. Instead, consistent with its original use, actifpTM was applied only to an already filtered set of high‐confidence models as an additional validation metric to confirm the robustness of top‐ranking predictions.

FoldSeek‐Multimer Cluster was used to group predicted protein complexes according to structural similarity, allowing identification of reproducible and biologically plausible interface patterns across independent models (Barrio‐Hernandez et al., 2023; Kim et al., 2025; van Kempen et al., 2024). The tool was installed and run locally via command‐line interface from the FoldSeek GitHub repository (accessed February 2025, available at https://github.com/steineggerlab/foldseek). Structural similarity between models was quantified using the superimposition‐free interface local distance difference test (lDDT) score, which measures how closely the interacting residues of two complexes align in three‐dimensional space. An interface lDDT threshold of 0.80 was used to define significant structural convergence.

### 2.4 New generation confidence scores

The interchain predicted TM‐score (ipTM) was used as a good indicator of predicted global reliability, with values ≥0.75 treated as high confidence, values between 0.60 and 0.75 treated as intermediate confidence, and values <0.60 considered low confidence (O'Reilly et al., 2023; Yin et al., 2022). Each dimer model generated in the screen was evaluated using multiple confidence scores. First, a weighted combination of ipTM and pTM from AlphaFold2 and AlphaFold3 predictions was calculated as 0.8 × ipTM + 0.2 × pTM. Scores above 0.80 were considered high confidence, values between 0.60 and 0.80 were considered intermediate, and a permissive threshold of 0.40 was used to include lower‐confidence interactions (Evans et al., 2021).

The ipTM evaluates interactions between chains rather than individual interface regions. To address this, we employed the ipSAE (Dunbrack, 2025). The ipsae.py script calculates ipSAE based entirely on interchain residue pairs with PAE(i,j) values below a defined cutoff. When no residue pairs meet this criterion, ipSAE returns 0.0. The script also computes two additional forms for comparison: ipSAE_d0chn and ipSAE_d0dom. ipSAE_d0chn uses the same PAE cutoff as ipSAE but defines d0 as the sum of the full‐length sequences of the two chains. ipSAE_d0dom calculates d0 from the number of residues in the two chains that have any interchain PAE values below the cutoff. Importantly, ipSAE does not require modifications to the AlphaFold codebase and relies solely on standard output files (e.g., JSON) from both AlphaFold2‐Multimer and AlphaFold3. A PAE cutoff of 10 Å and an ipSAE threshold of 0.30 have been suggested to distinguish likely true interactions from false positives (Dunbrack, 2025). For comparisons across ORP‐PIP complexes, we focus on ipSAE_d0dom rather than the standard ipSAE score because it normalizes by residues that participate in the interchain interface. This prevents differences in chain length or disordered regions in the ORP and PIP sequences from artificially lowering the confidence score, enabling a more accurate comparison of potentially conserved interfaces across structurally aligned models, which were subsequently analyzed with FoldSeek‐Multimer (Kim et al., 2025). Network visualization was performed in Gephi (Bastian et al., 2009), using clustering solely to highlight groups of SAC1‐ORP complexes with similar interfaces based on FoldSeek‐Multimer interface lDDT scores.

The ipSAE_d0dom score was applied to protein dimer models by downloading a Python script via its GitHub page (accessed February 2025, available at https://github.com/DunbrackLab/IPSAE). The PAE cutoff and the inter‐residue distance cutoff were both set to 15 Å, consistent with the recommended parameters of the script. Scores below 0.20 were considered indicative of false complexes, while scores above 0.40 were considered indicative of true complexes. ipSAE (interaction prediction Score from Aligned Errors) was applied to dimer models only, as it does not provide a global confidence measure for higher‐order assemblies.

The predicted DockQ score (pDockQ, a portmanteau combining the words “Docking” and “Quality”) was included as an additional metric calculated using the ipSAE script (Bryant et al., 2022; Zhu et al., 2023). Values above 0.23 were considered acceptable, scores between 0.23 and 0.48 indicated acceptable accuracy, 0.49–0.79 indicated medium accuracy, and scores of 0.80 or higher indicated high accuracy (Basu & Wallner, 2016; Collins et al., 2024).

Another confidence score used was the actual interface pTM (actifpTM), which is calculated by AlphaFold2‐based LocalColabFold during the model generation step (Varga et al., 2025). No formal threshold was defined but it was used to evaluate whether model confidence followed patterns similar to the weighted ipTM + pTM score. In this context, actifpTM values above 0.70 were considered indicative of the most reliable predictions, while values between 0.60 and 0.80 were considered intermediate. AlphaFold2 predictions in this study incorporated a refined confidence metric known as actual interface predicted TM‐score (actifpTM) (Varga et al., 2025). This enhancement addresses a key limitation of the original interchain predicted TM‐score (ipTM), which struggles to accurately assess interaction interfaces in the presence of long intrinsically disordered regions or non‐interacting flanking segments, features commonly omitted in crystal structures but often present in full‐length sequences. While ipTM has been applied in docking studies involving peptide‐protein complexes (Bret et al., 2024; Lee et al., 2024; Teufel et al., 2023), it was originally trained on structured PDB entries and is sensitive to sequence length, treating all residues equally regardless of their involvement in the interface. As a result, inclusion of disordered or accessory regions can lead to substantial reductions in ipTM scores, even when the core interaction is well predicted.

To mitigate this, actifpTM modifies the ipTM calculation by masking non‐interacting regions and reweighting residue pairs based on predicted distance probabilities. This makes the score more reflective of true interface quality, independent of overall sequence length or structural disorder. Two components are essential for calculating the actifpTM score, both related to contact maps derived from distograms generated during model prediction. Note that a distogram is required to calculate the contact map. AlphaFold3 does not provide distograms, so it cannot calculate this score from a model. Second, probability maps from the PAE matrix are needed. These are not produced in the AlphaFold3 protocol (Abramson et al., 2024).

### 2.5 Protein structure analysis

To assess the potential functional relevance of candidate interfaces, we employed the ConSurf web server (accessed March 2024, available at https://consurf.tau.ac.il) to identify evolutionarily conserved regions (Landau et al., 2005). This approach allowed us to highlight residues likely to be functionally important or structurally constrained. Homologous sequences were identified from the UniRef90 database, and a MSA was generated using ConSurf's Bayesian inference approach with the default maximum of 150 sequences. Residue‐specific evolutionary conservation scores were calculated and mapped onto the protein structure using ConSurf's nine‐grade color‐coding system, in which scores range from 1 (highly variable positions) to 9 (highly conserved positions), providing a qualitative representation of evolutionary constraints across the protein surface. Sequence‐based conservation analysis was performed using ProbCons with default parameters (two consistency iterations, 100 iterative refinement steps, no pre‐training). Alignments were visualized using locally installed Jalview software (Do et al., 2005; Troshin et al., 2011) and exported in CLUSTALW format, for which the pairwise percentage identity was reported (Tables S1, S2).

In parallel, we used the PeSTo web server (Krapp et al., 2023), a parameter‐free geometric deep learning tool, to predict protein–protein interaction interfaces (accessed April 2024, available at https://pesto.epfl.ch). By integrating ConSurf conservation data with PeSTo's interface predictions, we aimed to pinpoint conserved regions likely involved in mediating interactions between the proteins of interest.

Electrostatic surface potentials were computed using the APBS (Adaptive Poisson‐Boltzmann Solver) plugin implemented in PyMOL (accessed March 2024, available at https://www.poissonboltzmann.org) with default parameters (Jurrus et al., 2018; Schrödinger & DeLano, 2020). Electrostatic potentials were mapped onto the molecular surface and visualized using a color scale ranging from −5 to +5 kT/e, corresponding to negative and positive electrostatic potential values, respectively. Calculations were performed under standard near‐physiological conditions, with solvent and solute dielectric constants, ionic strength, temperature, and grid dimensions automatically assigned based on the protein size.

The CoCoNat deep‐learning web server built by the Bologna Biocomputing Group (accessed September 2024, https://coconat.biocomp.unibo.it) was used to analyze the AlphaFold3‐predicted ORP10‐ORP11 dimer interaction by uploading the PDB model (Madeo et al., 2023). Amphipathicity of SAC1 helices was assessed using the HeliQuest web server (Gautier et al., 2008) by submitting the protein sequences of three prominent α‐helical regions, residues 101–137, 424–450, and 464–500 (accessed September 2024, https://heliquest.ipmc.cnrs.fr/index.html).

### 2.6 Building protein‐membrane systems with CHARMM‐GUI

Protein‐membrane systems were prepared using CHARMM‐GUI (accessed May 2025, available at https://charmm-gui.org), employing the Bilayer Builder module within Membrane Builder (Jo et al., 2007; Jo et al., 2008; Wu et al., 2014). Protein coordinates were uploaded as membrane‐oriented structures. To define their orientation relative to the lipid bilayer, structures were first analyzed using the PPM 3.0 server with default parameters (Lomize et al., 2012; Lomize et al., 2022). System size was then defined using a heterogeneous rectangular lipid bilayer, specifying a water thickness of 22.5 Å and a fixed ratio of lipid components, without generation of pore water. No salt was added. The membrane system was constructed using the replacement method, without applying the ion placement procedure (Lee et al., 2016). CHARMM‐GUI was used exclusively for system construction, and no molecular dynamics simulations or equilibration steps were performed in this work. Membrane size and composition data are reported in Tables S3, S4.

### 2.7 Subcellular localization analysis from proteomics data

We consulted the Human Cell Map non‐negative matrix factorization (NMF) outputs (Go et al., 2021), Prolocate (Jadot et al., 2017) and Map of the Cell (Itzhak et al., 2016) databases for our proteins of interest. The data we extracted from Prolocate came from Experiment B, conducted on liver tissue of fed rats. When proteins of interest were not detected under these normal conditions, we relied on annotations from Experiment A on fasted rats.

## 3 RESULTS

### 3.1 New generation scores reveal ORP‐PIP interactions

**FIGURE 1:**
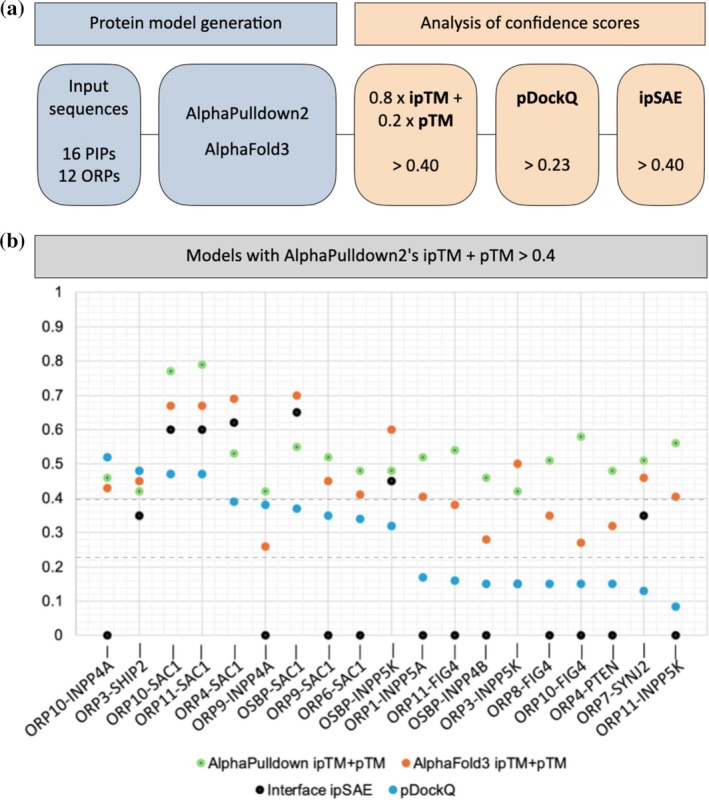
Confidence score‐based ranking of ORP‐PIP dimer models. (a) Structural screening protocol for ORP‐PIP interactions. We employed AlphaFold3 and AlphaPulldown2. “ipTM+pTM” represents the weighted confidence score, calculated as 0.8 × ipTM +0.2 × pTM. “ipSAE” represents the ipSAE_d0dom score. High‐scoring models were validated in further ways as described in the text. (b) Graph of AlphaFold3 and AlphaPulldown2 models from the ORP dataset. Twelve human paralogues were used as baits against 16 PIPs to predict protein–protein interactions. The 19 highest scoring models are shown by using an AlphaPulldown2 weighted ipTM+pTM threshold of >0.40. Two gray dashed lines indicate thresholds at y = 0.40 (for ipTM + pTM and ipSAE) and y = 0.23 (for pDockQ).

We systematically screened 12 human ORPs for potential interactions with 16 PIPs implicated in PI turnover at ER‐PM and ER‐Golgi junctions. Our goal was to identify potential interactions between PIPs and lipid transfer proteins and explore how structure and localization data converge to suggest functional partnerships. To this end, we created a protocol for systematic structural screening (Figure 1a). This effort produced a structural interaction dataset of nearly a thousand AlphaPulldown2 models. The top‐scoring structures were identified, and their sequences were independently modeled and rescored using AlphaFold3. Despite substantial variability between model confidence scores across methods, a subset of interactions consistently scored above threshold and showed spatial interface coherence, suggesting genuine biological relevance.

In an initial AlphaPulldown2 screen we applied a permissive weighted confidence ipTM+pTM threshold (>0.40) to maximize sensitivity for our models of interest. Figure 1b presents the confidence scores for the top‐ranking models, where each model (x‐axis) is associated with multiple scoring metrics plotted along the y‐axis. Horizontal dashed lines indicate the applied confidence thresholds, with 19 models exceeding the ipTM + pTM cutoff (>0.40). Several models scored highly in one system but not the other, like in the case of pairs involving the phosphatase INPP4A. These discrepancies highlight the limitations of relying on a single confidence metric.

Those models scoring high with ipSAE also showed high‐confidence values in AlphaFold3 (shown in green), suggesting the ipSAE score might correlate better with the more holistic confidence estimation in AlphaFold3 (Figure 1b). Still, we must remain cautious, particularly with the weighted ipTM + pTM metric, which penalizes models with low pTM, which can result from disordered regions present in some of the proteins studied here. This is especially evident in the ORP protein chains, where flexible linkers reduce global confidence despite strong local interfaces.

Although a few models exceeded individual confidence thresholds (Figure 1b), only SAC1‐ORP complexes were retained for further analysis later in this study because they consistently exhibited interface conservation across predictions. The ORP7‐SYNJ2 model was excluded due to a pDockQ score below the confidence cutoff (<0.23), while OSBP‐INPP5K models were explicitly evaluated using FoldSeek‐Multimer across all ORP‐INPP5K predictions. No clustering or conserved interface was detected based on interface lDDT in the next section, indicating a lack of reproducible interaction geometry. Consequently, these models did not meet the combined confidence and conservation criteria applied throughout this study and were not pursued further.

### 3.2 Interface clustering reveals SAC1 can interact with ORP paralogues

While ORPs vary widely in domain architecture and organelle specificity according to the literature (Du et al., 2011; Kawasaki et al., 2022; Murphy & Levine, 2016; Olkkonen & Li, 2013), many share a similar orientation when binding to SAC1 in our models. Therefore, we performed systematic clustering of predicted complexes based on interface similarity rather than global structure or sequence identity. This approach uncovered a conserved SAC1‐binding mode across multiple ORP paralogues. To examine any inconsistencies between AlphaPulldown2 and AlphaFold3 modeling and scoring, we introduced FoldSeek‐Multimer clustering to cross‐check interfaces predicted independently by the two tools. Since our top‐scoring models involved protein pairs containing INPP5K or SAC1, we focused on this subset and trimmed the ORP models to their ORD domains after observing that this region consistently formed the primary interface across the dataset.

**FIGURE 2:**
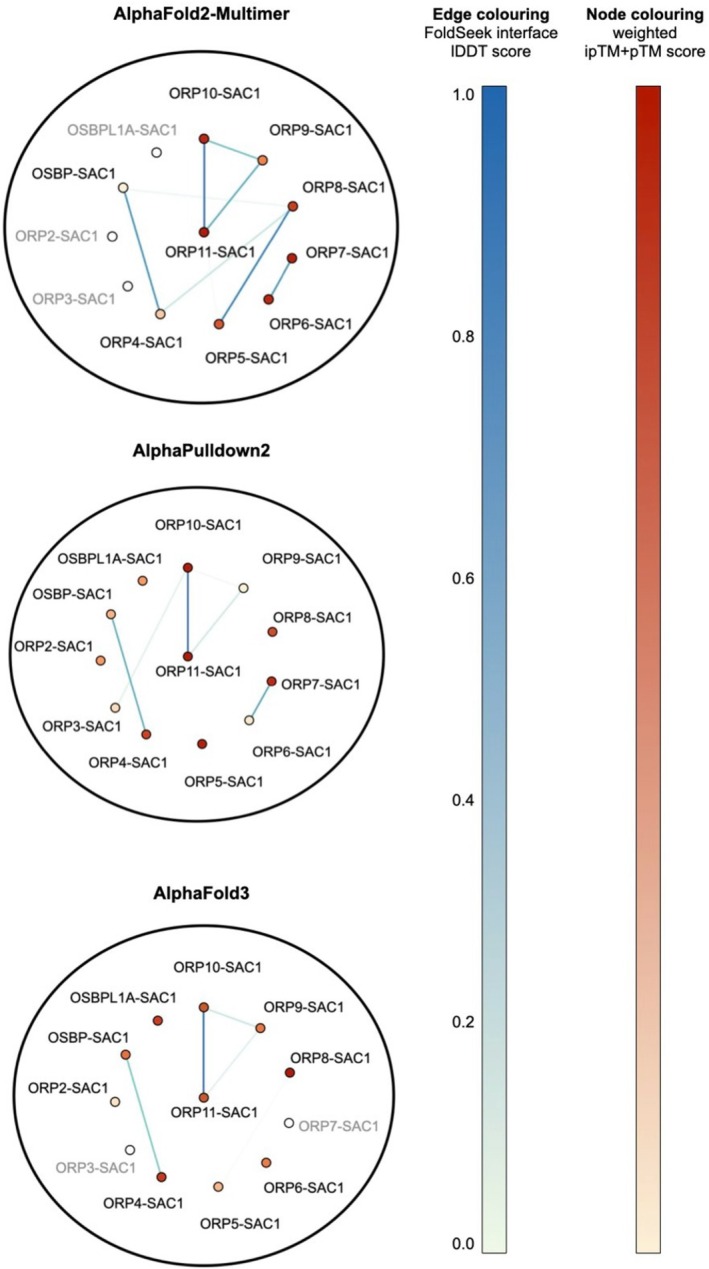
Network clustering of SAC1‐ORP protein pairs based on interface similarity using FoldSeek‐Multimer. Three networks were generated from models produced by AlphaFold2‐Multimer, AlphaPulldown2, and AlphaFold3. Models were trimmed to the ORP ORD domain, while SAC1 was kept full‐length. Each node represents a model with ipTM + pTM >0.40. Edges reflect interface lDDT similarity, with thickness and color indicating similarity strength and ipTM+pTM scores (blue and orange scales, respectively). Networks were filtered for FoldSeek interface lDDT >0.80. Gray text boxes represent complexes that were not modeled in AlphaFold2‐Multimer or AlphaFold3 as the same protein pairs in AlphaPulldown2 had a weighted ipTM+pTM confidence score <0.40. Layout: Fruchterman‐Reingold algorithm; analyzed in Gephi (Bastian et al., 2009).

Using the FoldSeek‐Multimer cluster protocol, we calculated interface‐local lDDT scores for predicted complexes involving all 12 ORPs and our panel of 16 PI phosphatases. The interface lDDT metric quantifies local interface similarity without requiring global structural superposition, unlike TM‐align. To ensure we captured meaningful structural convergence, we set an arbitrary interface similarity cutoff (interface lDDT >0.80), similar to that used in global alignments with the TM‐score (Pereira et al., 2025). This threshold does not assume identical binding modes for all complexes but provides a consistent criterion to highlight conserved interfaces. At this cutoff, only models involving SAC1 retained strong clustering, suggesting a structurally conserved binding interface not observed in predicted complexes with other phosphatases (Figure 2). No other phosphatase engaged in complexes that were similar within its own cluster or to the SAC1‐ORP cluster, indicating that the observed clustering is specific to the SAC1‐ORP interactions.

We visualized the similarity network using a Fruchterman‐Reingold layout, where each node represents a model and edges reflect interface similarity scores. Models formed a scattered network. If we apply an lDDT >0.80 threshold, a coherent subcluster emerges, consisting of SAC1 bound to ORP10 and ORP11. This refined cluster is consistent across the different versions of AlphaFold we used: the nodes indicate strong ipTM + pTM scores and the edges highlight consistent interface geometries between the SAC domain of SAC1 and the ORD domain of ORPs. Importantly, we took a conservative approach in interpreting the data, focusing only on models where both interface similarity and confidence metrics were significant. A level of agreement between AlphaFold3, AlphaFold2‐Multimer, and AlphaPulldown2 models for individual protein pairs was seen only for the SAC1‐ORP11 complex. These results underscore the added value of interface‐based metrics in large‐scale structural screens.

We further illustrate the similarities and differences between AlphaPulldown and AlphaFold3 in modeling the ORP9‐SAC1 (Figure S1) and ORP10‐SAC1 complexes (Figure S2). The main sources of variation are the coiled‐coil region and the relative orientation of the PH domain, both of which shift substantially between modeling approaches. When these regions are removed, the remaining structures reveal clear structural similarities across protein pairs, and this is reflected by their sequence alignments (Table S1). Accordingly, Figure 2 includes cross‐ORP and cross‐program comparisons (AlphaPulldown versus AlphaFold3) showing that some models appear visually similar, particularly at the interface, yet their local interface lDDT scores occasionally fall just below the 0.80 threshold. Full‐length protein alignments showed lower interface lDDT compared to alignments restricted to the ORD domains.

Using this trimming strategy, several previously unreported ORP‐SAC1 complexes with similar interface architectures emerged, including ORP4‐SAC1 and OSBP‐SAC1 (pairwise ORD alignment 67.89%), ORP10‐SAC1 and ORP11‐SAC1 (pairwise ORD alignment 71.10%), and ORP9‐SAC1 and ORP10‐SAC1 (pairwise ORD alignment 55.78%), as shown in Table S2. This is in fact reflected by their sequence alignments. These similarities stand in contrast to the lower sequence identity observed when full‐length proteins are compared, for example 54.38% between ORP10 and ORP11 and 37.25% between ORP10 and ORP9, underscoring that overall protein alignment is substantially lower than the ORD‐focused alignments. Notably, the highest ORD similarity is observed between ORP11 and ORP10, reaching 71.10%, and ORP11‐SAC1 models also scored more confidently by AlphaFold. All of these interaction patterns were consistently recovered across all three modeling approaches. While we do not analyze each complex in detail, these results demonstrate that trimming ORP models to their ORD domains is a practical and effective way to leverage FoldSeek‐Multimer and expand the landscape of plausible ORP‐SAC1 interactions.

We also observed ORD structural similarities for ORP6‐SAC1 and ORP7‐SAC1 models between AlphaPulldown2 (interface lDDT ~0.93) and AlphaFold2‐Multimer (interface lDDT ~0.85), and the same goes for the ORP5‐SAC1 and ORP8‐SAC1 models generated by AlphaFold2‐Multimer (interface lDDT ~0.95). These predictions, based solely on the ORD domain of ORPs, suggest a conserved binding interface. We focused on ORP11‐SAC1 above other pairs, and subsequently on its similarities with ORP9‐SAC1 and ORP10‐SAC1, since the other AlphaFold models show greater uncertainty in the orientation of PH and coiled‐coil domains, whereas ORP11 consistently displays a clear SAC1 binding interface across AlphaFold methods. Notably, the predicted orientation of SAC1's ER‐embedded TM domain in contact with ORP4, ORP6, and ORP7 coiled‐coil domains is biologically implausible, and we do not consider it a valid secondary binding site.

Overall, a comparison of the most structurally similar interfaces (Figure 2) with the sequence identity matrix of ORD domains (Table S2) shows a correlation. The strongest linkages in Figure 2 generally correspond to high sequence identities in a family of significant sequence diversity (Tables S1, S2). For example, ORPs10 and 11 share 54% sequence identity in their ORD domains, while the figure of OSBP and ORP4 is 68%. This correlation is not unexpected, but demonstrates that there appears to be no sign of convergent evolution toward similar interfaces among distant relatives.

### 3.3 ORP11‐SAC1 in a membrane system

We note that the emphasis on ORP11‐SAC1 in this study is not because of more consistent domain‐level arrangements, but rather because it produced the highest‐confidence models in AlphaFold predictions and because an ORP‐SAC1 interaction has been previously observed in yeast via crosslinking (Stefan et al., 2011). From our broad screen of ORP‐phosphatase interactions, the ORP11‐SAC1 complex emerged as a particularly robust pairing, with consistent high‐confidence models across AlphaFold2, AlphaFold3, and FoldSeek‐Multimer clustering. Building on this, we manually superimposed the AlphaPulldown2 and AlphaFold3 models of ORP11‐SAC1, both of which score above 0.80 in interface lDDT according to FoldSeek‐Multimer to assess their structural agreement in more detail.

**FIGURE 3:**
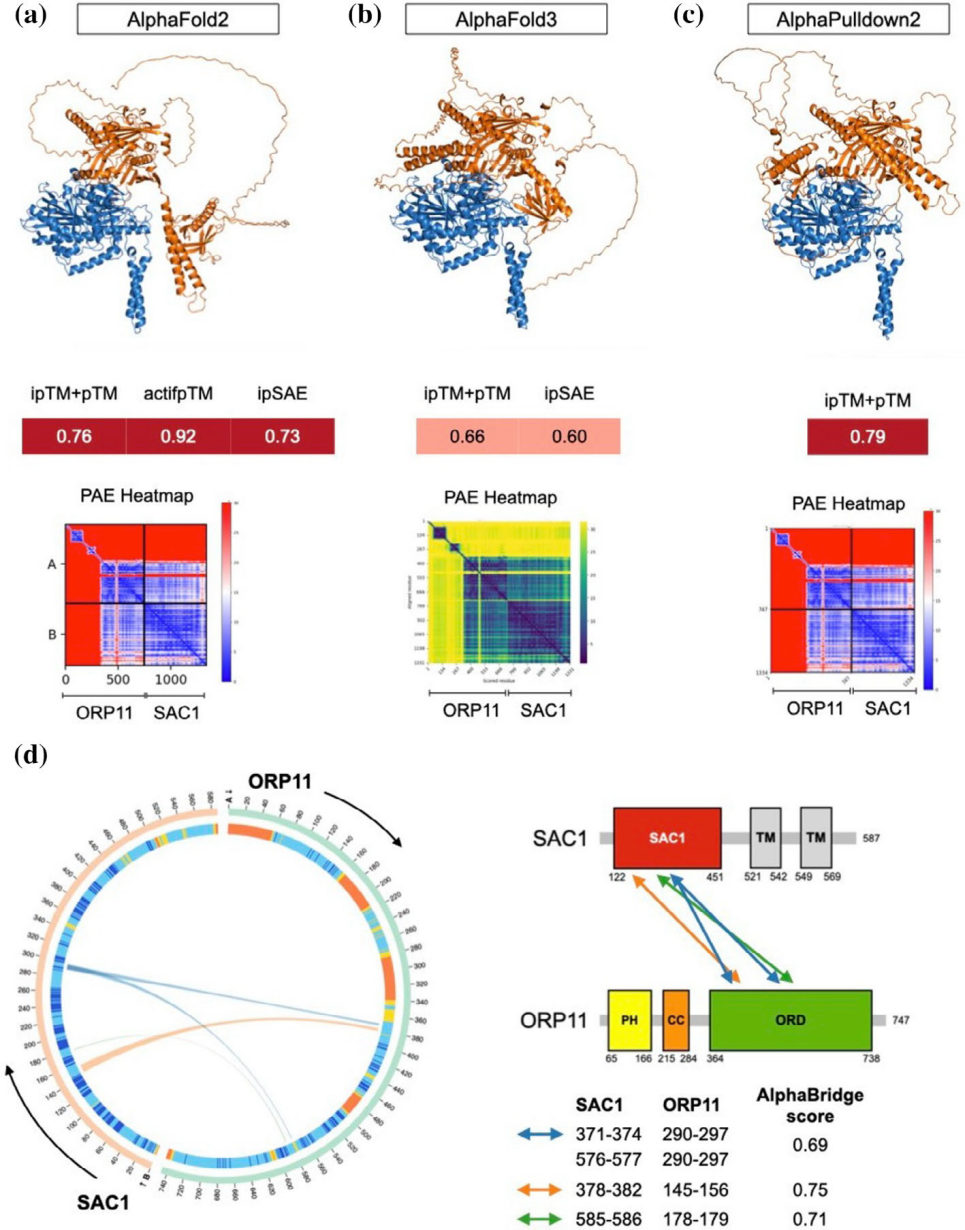
Structural superimposition of (a) AlphaFold2‐Multimer, (b) AlphaFold3, (c) and AlphaPulldown2 models for the ORP11‐SAC1 complex. PAE heatmaps and confidence scores for each model are shown right below. “ipTM+pTM” represents the weighted confidence score, calculated as 0.8 × ipTM +0.2 × pTM. “ipSAE” represents the ipSAE_d0dom score. (d) AlphaBridge results for the AlphaFold3 ORP11‐SAC1 model (Álvarez‐Salmoral et al., 2024). Left: Chord diagram showing residue‐residue interactions between ORP11 and SAC1 along a circular representation of their sequences, colored by per‐residue pLDDT confidence scores (AlphaFold color scheme; credit: Konstantin Korotkov). Lines connecting residues indicate contacts at the interface and are colored according to the AlphaBridge interaction score, reflecting interaction strength. Right: Protein domain maps of ORP11 and SAC1, with three arrows (blue, orange, and green) highlighting the regions involved in the interaction.

We noticed that while the core interface between the SAC1 catalytic domain and the ORP11 ORD domain aligns closely across models, notable differences appear elsewhere (Figure 3a–c). The coiled‐coil α‐helices of ORP11 occupy different positions in each model, and the PH domain undergoes a complete shift in orientation. These discrepancies align with regions of lower predicted alignment confidence in the PAE heatmaps, particularly those corresponding to the sequences of the N‐terminal half of ORP11 domains (Figure 3a–c). In contrast, the core SAC1‐ORD interface lies in a high‐confidence region, reinforcing the consistency of this interaction. Coloring the models by pLDDT shows that AlphaPulldown2 and AlphaFold3 differ in this same region, consistent with the lower predicted alignment confidence in the PAE heatmaps just discussed (Figure S3). While both AlphaPulldown2 and AlphaFold3 assign high pLDDT scores to the core interface, AlphaPulldown2 exhibits substantially lower confidence in the coiled‐coil and PH domains compared to AlphaFold3 (Figure S3). This suggests that AlphaFold3 may provide a more reliable model overall, at least for these flexible or peripheral regions. Despite these local uncertainties, the main SAC1‐ORD interface remains structurally the same between methods, underscoring the robustness of this interaction even when peripheral domain positions diverge. The SAC1‐ORD interface comprises three key interactions as reported by AlphaBridge results for the AlphaFold3 model (Figure 3d) and more than 90% of interacting residues of SAC1 and ORP11's ORD domain are shared between models generated by AlphaFold3, AlphaFold2‐Multimer, and AlphaPulldown2.

Having established a preference for the AlphaFold3 model of ORP11‐SAC1 based on the near‐ideal positioning of the PH domain adjacent to the putative membrane plane where SAC1 resides, we next examined the position of the PH domain in SAC1 (Figure S3). In the AlphaFold3 model, the PH domain is oriented toward where a membrane would be ideally. In contrast, the AlphaPulldown2 model positions the PH domain closer to the SAC domain, possibly indicating an alternate, transient, or less reliable interface. Despite these differences, both models share most key interface residues that overlap between SAC1 and the ORP11 ORD domain.

**FIGURE 4:**
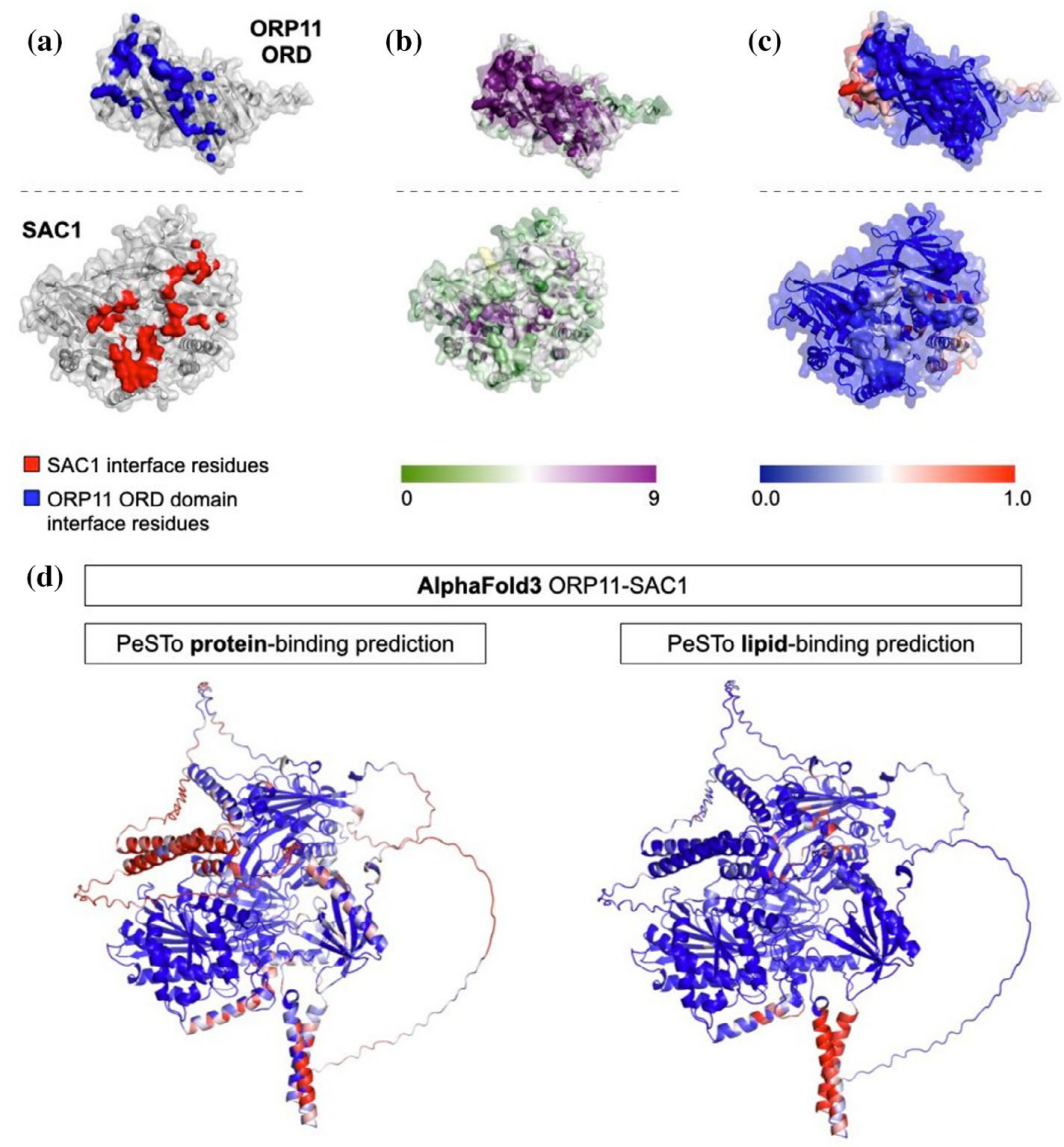
Functional characterization of the ORP11 ORD‐SAC1 interface. (a) Open‐book view of the ORP11 ORD‐SAC1 interface, highlighting key interacting residues. (b) Same view colored by evolutionary conservation from ConSurf. (c) Same view showing PeSTo‐predicted protein–protein binding residues. (d) Full complex view (same orientation as in Figure 3), with PeSTo predictions overlaid: Protein–protein binding residues on the left and lipid‐binding residues on the right.

To assess the potential functional relevance of the ORP11‐SAC1 interface, we visualized the predicted interface using an open‐book view (Figure 4a). Conservation analysis with ConSurf (Figure 4b) indicated that several interface residues, particularly on ORP11, are evolutionarily conserved, with some conserved residues present on SAC1. Protein–protein interaction predictions from PeSTo (Figure 4c) supported these findings. Notably, the following residues on ORP11 were identified by both ConSurf and PeSTo as likely interface sites: S368, V369, H372, L373, and Q376‐D382. For SAC1, overlapping predictions included Y482 and K483, though overall agreement between the two methods was lower for this protein. There is a high‐scoring helix in SAC1 predicted to interact with both proteins and lipids (Figure 4d). The helix runs parallel to the membrane bilayer, and when SAC1 is embedded at the ER, its proximity to the upper leaflet likely occludes any potential protein binding sites.

**FIGURE 5:**
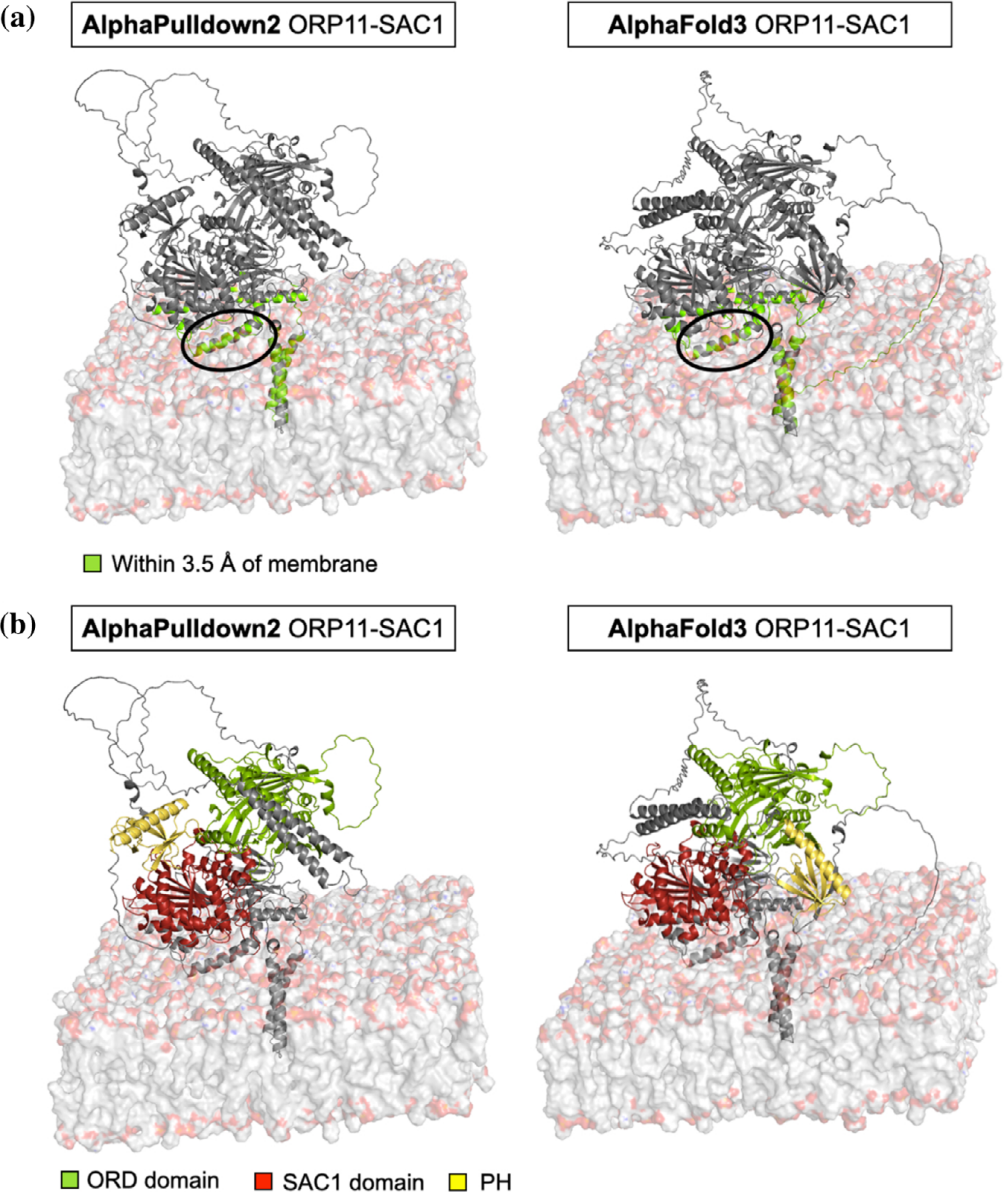
ORP11‐SAC1 in a membrane system. (a) AlphaFold3 and AlphaPulldown2 models of ORP11‐SAC1 inside the membrane. The protein complex structure was oriented based on the bilayer plane using the PPM 3.0 server and then embedded in the ER‐like membrane generated using CHARMM‐GUI. Residues within 3.5 Å of the upper membrane leaflet are colored green, indicating potential membrane contacts. Circled is the helix in SAC1 that we report to be amphipathic. (b) The same models colored by domain architecture to highlight structural differences. In the AlphaFold3 model, the PH domain (in yellow) of ORP11 is positioned closer to the membrane, whereas in the AlphaPulldown2 model, the PH domain interacts directly with the SAC domain of SAC1.

We used CHARMM‐GUI to assess the compatibility of our models with membrane insertion (Lomize et al., 2012) after orienting the protein structure correctly using the PPM 3.0 tool (Lomize et al., 2022). (Figure 5a). Residues within 3.5 Å of the membrane surface are highlighted in green. In the AlphaFold3 model, the PH domain of ORP11 sits just above the membrane, poised for lipid engagement, while the ORD domain contacts SAC1 laterally. In contrast, the AlphaPulldown2 model positions the PH domain away from the membrane, possibly reflecting an alternate or pre‐bound state. Nevertheless, in both the AlphaFold3 and AlphaPulldown2 models, the PH domain remains close enough to the membrane for lipid binding without being occluded or hindered by SAC1 (Figure 5b).

We examined the α‐helices in SAC1 that lie near the upper leaflet of the bilayer to assess their amphipathic character using HeliQuest (Gautier et al., 2008). Only the segment “FLAMLNHVLNVDGFYFST” (residues 119–135) shows notable hydrophobicity (0.77). This region (marked with a circle in Figure 5a) is located very close to the membrane and may also contribute to the stabilization of the ORP‐SAC1 pair complex (Figure S4). However, AlphaFold predicts that about half of these residues form a curved loop extending away from the membrane, suggesting that this helix may simply require model refinement in AlphaFold rather than forming a stable amphipathic region above the curved bilayer.

### 3.4 Higher‐order ORP dimeric assemblies explored

Following our analysis of ORP‐phosphatase interactions, we turned our attention to the possibility of higher‐order assemblies involving ORP9, ORP10, and ORP11. This direction was motivated by a recent study reporting that ORP9 and ORP11 form heterodimers (Cabukusta et al., 2024). We sought to determine whether state‐of‐the‐art structure prediction tools could recapitulate this dimer interface, whether such a dimer could be predicted with any degree of confidence, and whether SAC1 might be incorporated into these higher‐order assemblies.

We summarize here the results from AlphaFold3 and AlphaFold2‐Multimer predictions for the ORP10‐ORP11 dimer (Figures S5, S6). Overall, confidence scores were low across both tools. Importantly, the predicted dimer interface differed substantially between AlphaFold3 and AlphaFold2, highlighting a lack of convergence. The AlphaFold3 model positioned the PH domains of both ORPs in close proximity and suggested a potential coiled‐coil‐mediated interaction. The PAE heatmap showed medium‐confidence contacts at this interface, but corresponding pLDDT scores were uniformly low (Figure 4c), suggesting significant uncertainty or potential disorder in this region. The AlphaFold2 model also showed little overall confidence, with the actifpTM score clearly reflecting low interface reliability (Figure S6).

Next, we tested whether SAC1 could be integrated into this putative ORP10‐ORP11 dimer. Adding a single SAC1 molecule in the AlphaFold3 prediction resulted in severe steric clashes between the ORD domains of ORP10 and ORP11 (Figure S7A), ruling out this configuration. In contrast, AlphaFold2‐Multimer was able to place SAC1 without clashes (Figure S7B, C). This model showed one ORD domain from ORP10 engaging SAC1 through the interface we have consistently seen in previous models, while the second ORD domain from ORP11 approached SAC1 from a lateral angle, forming additional contacts. The actifpTM score here indicated moderate to high confidence in this asymmetric interaction. Still, this model should be interpreted with caution, as only AlphaFold2 predicted it without generating steric conflicts, and the interface was not replicated by AlphaFold3.

Finally, we examined a tetrameric configuration where two SAC1 molecules bind an ORP10‐ORP11 dimer, testing whether SAC1 might interact with each ORP separately. The resulting models, shown in Figure S8, failed to support this architecture. Confidence scores dropped again, and both AlphaFold3 and AlphaFold2 produced structurally incompatible configurations. In AlphaFold3 and AlphaFold2, the TM domains of SAC1 were misoriented relative to a hypothetical membrane bilayer. PAE heatmaps highlighted widespread uncertainty in domain positioning and overall spatial arrangement, undermining the plausibility of this tetrameric complex.

We next turned to the ORP9‐ORP11 pair, which has been reported to form dimers (Cabukusta et al., 2024). Both AlphaFold3 and AlphaFold2‐Multimer predict the same mode of interaction, identifying a shared interface between their coiled‐coil domains, although they differ in overall domain arrangement and confidence metrics. In AlphaFold3 models (Figure S9A), the heterodimer achieves a high ipSAE score of 0.85, with the PAE heatmap indicating a well‐defined coiled‐coil interaction. Inclusion of a single SAC1 chain results in apparent contacts between SAC1's TM domain and residues in ORP9's ORD domain (Figure S9B), a biologically implausible interaction given that this region is membrane‐embedded. Adding a second SAC1 chain to form a tetramer with ORP9‐ORP11 restores symmetry and allows SAC1 TM domains to dimerise (Figure S9C). Here, pLDDT and weighted ipTM+pTM scores are lower than previous models, but ipSAE and pDockQ remain above 0.50.

Crucially, comparison with AlphaFold2‐Multimer models (Figure S10) confirms that the coiled‐coil interface between ORP9 and ORP11 is preserved, consistent with what is experimentally observed in the literature (He et al., 2023; Zhou et al., 2010). The actifpTM scores in these models are uniformly high (>0.90). Minor misorientation of SAC1 TM domains in some multi‐chain models (Figure S10C) is likely inconsequential, as these domains are connected to the SAC domain by a flexible linker. Notably, in the AlphaFold3 PAE plots, additional low‐PAE regions corresponding to the PH domains are observed. These may reflect spurious contacts rather than biologically relevant protein–protein interactions, as the ORP PH domains are primarily lipid‐binding modules (He et al., 2023; Kawasaki et al., 2022; Nakatsu & Kawasaki, 2021).

## 4 DISCUSSION

Interpreting predicted protein–protein interactions requires more than structural plausibility; it demands a biologically grounded framework that filters out artifacts and elevates meaningful contacts. In this study, we only consider an interaction to be confidently predicted when multiple independent lines of evidence align. High interface quality, measured primarily through actifpTM scores and supported cautiously by ipSAE, is a first requirement. Good scores alone are insufficient. Predictions must show compatible subcellular localization. Interactions between ER‐ and cytosol‐localized proteins are plausible for peripheral or integral ER proteins only if the interaction surface maps to the cytoplasmic region; luminal ER proteins, by contrast, are topologically isolated and unlikely to engage cytosolic partners. This topological consideration is an important factor that is often overlooked but critical for filtering biologically meaningful interactions. Structural plausibility is equally critical, particularly for membrane‐associated complexes: the predicted interface must not violate known membrane topologies or orientations. Finally, consistency across models between AlphaFold2 and AlphaFold3, and/or among homologs adds crucial support. pLDDT and congruent interface geometries across models indicate structural reliability. By this stringent standard, only a minority of predictions are accepted. The ORP11‐SAC1 complex is one such case, satisfying all criteria and exhibiting high‐confidence interface geometry across the methods used.

These criteria are not arbitrarily conservative; they reflect fundamental architectural and methodological differences between AlphaFold versions and protocols. AlphaPulldown2 builds on AlphaFold2‐Multimer, whose Evoformer module extracts co‐evolutionary information from MSAs and refines pairwise residue representations through transformer‐based iterations. The Evoformer is effective when deep MSAs are available but suffers in sparse evolutionary landscapes (Yang et al., 2023). AlphaFold3 is very different. Its multimodal diffusion‐based architecture accepts not only sequence, MSA input and structural templates, but also ligands and ions (Abramson et al., 2024). As a result, AlphaFold3 can succeed where AlphaFold2 and AlphaPulldown2 fail, but also diverge at times, producing markedly different interfaces, loop conformations, and subunit orientations even from the same input sequences.

### 4.1 Assessing interactions with new generation confidence scores

Given the considerations above, it is important to note that model confidence scores from different methods (i.e., ipSAE vs. actifpTM) can vary substantially and do not always agree. While the scores all operate on a nominal 0–1 scale, they are not always directly comparable. Independent studies have established empirical thresholds for model confidence for one of the metrics we use. pDockQ scores above 0.23 are predictive of likely interactions (Bryant et al., 2022; Zhu et al., 2023), among which scores of ≥0.80 indicate high‐accuracy models, 0.49–0.79 correspond to medium accuracy, and 0.23–0.48 are considered acceptable (Basu & Wallner, 2016). Our best models in this study fall within the 0.30–0.50 range, consistent with acceptable to medium‐quality predictions. While pDockQ is not a definitive standard for model assessment, and arguably no such gold‐standard metric exists yet, it still provides a useful frame of reference.

The ipSAE score is another key metric we used, and it is not limited to interface residues (Dunbrack, 2025). Instead, it is based on all inter‐chain residue pairs with PAE(i,j) values below the chosen PAE cutoff. The interface distance parameter in the implementation is used only to report the number of residues meeting both the PAE and distance criteria, but it does not contribute to the ipSAE value itself. The score defaults to zero only when there are no inter‐chain residue pairs below the cutoff, that is, when AlphaFold predicts no confident relative positioning of residues across chains. This feature differentiates ipSAE from ipTM and related scores, which rarely approach zero because they consider all residue pairs regardless of confidence.

The ipSAE score has only recently been introduced and, while increasingly used (Abdelkareem et al., 2025; Catoiu et al., 2025; Mathew et al., 2025), has not yet been widely benchmarked by groups independent of its developers. In its original report, benchmarking on sets of true and false complexes showed that almost all false complexes scored below 0.20, while true complexes with correct structural models generally scored above 0.50, with few intermediate values (Dunbrack, 2025). In our dataset, the highest ipSAE values (≥0.60, for the ipSA_d0dom score employed here) were observed in complexes involving the phosphatidylinositol 3‐ and 4‐phosphatase SAC1, placing them above the benchmarked threshold.

### 4.2 Biological implications of ORP‐PIP interactions

A central point of this study is that structure prediction alone, especially from a single method, is often insufficient to infer functional interactions. To strengthen our findings, we incorporated subcellular localisation data from the Human Cell Map (Go et al., 2021), Prolocate (Jadot et al., 2017) and Map of the Cell (Itzhak et al., 2016) databases. The Human Cell Map, based on BioID rather than fractionation, captures proximity relationships instead of steady‐state distributions. This can place proteins at transient or peripheral contact sites and, in our case, highlights ORP2 and ORP8 at the nuclear outer membrane‐ER network, representing a potentially new localisation (Figure S11A). ORP3 and INPPL1 (SHIP2) are assigned to the PM, while a substantial fraction of the remaining proteins of interest is placed at early recycling endosomes (Figure S11A). This likely reflects the proximity‐labeling approach of the Human Cell Map, where vesicular trafficking hubs are frequently captured because of the high connectivity of endosomal compartments (Go et al., 2021).

The Map of the Cell database was created using HeLa cells rather than rat liver. Like Prolocate, it is based on subcellular fractionation but in a human cancer cell line. HeLa cells have an abnormal number of chromosomes (aneuploidy) and extensive genomic rearrangements. This genomic instability alters gene regulation and can directly impact the abundance and localization of many proteins and the stoichiometry of protein complexes compared to non‐cancerous cells. Map of the Cell annotations are less comprehensive, with many proteins missing localization assignments. Only SAC1, ORP3, and ORP10 are assigned to the ER, and ORP9 together with INPP5A are placed at the PM (Figure S11B). Overall, the discrepancies across datasets arise from the methodological contrast between proximity‐labeling (Human Cell Map) and biochemical fractionation (Map of the Cell). As a result, each method can highlight different protein localizations.

The Prolocate dataset derives from two experiments on rat liver, one from fasted and one from fed animals (Figure S11C). The data from Prolocate comes from two experiments: one conducted on fasted rats and one on fed rats. As a result, experiment A, which was performed on fasted rats, does not reflect conditions that are fully comparable to those obtained under healthy and normal conditions. Here, we review examples of Prolocate fractionation data from rat liver that illustrate how subcellular localisation can inform potential protein–protein interactions: (1) SAC1 and INPP5K are reported to localize at least partially to the ER, supporting the feasibility of their interaction with ER‐tethered ORPs; (2) ORP11 and SAC1 are found in overlapping ER‐ and cytosol‐enriched fractions; (3) INPP5K and SYNJ2 show partial localisation to ER‐PM junctions, possibly through interactions with less well‐characterized ER‐anchored partners beyond the canonical recruiter ARL6IP1 (Dong et al., 2018); (4) most ORPs are cytosolic, consistent with their role as lipid shuttles, but exceptions include ORP2 at the Golgi and ORP5 and ORP8 at the ER (Figure S11C).

The biological relevance of the ORP11‐SAC1 interaction is further underscored by the absence of FFAT motifs in ORP11, ORP10, ORP5, and ORP8. FFAT motifs typically mediate ER anchoring via interaction with VAPA/B (Du et al., 2011; Kawasaki et al., 2022; Murphy & Levine, 2016). In these FFAT‐lacking ORPs, SAC1 or other ER‐resident partners may substitute as recruitment factors. This mechanism is consistent with models proposed for OSBP‐ and ORP5/8‐mediated transport, where PI4P is transferred to the ER and dephosphorylated by SAC1 to drive lipid exchange against a concentration gradient (Del Bel & Brill, 2018; Zewe et al., 2018). In these models discussed by other groups, SAC1 acts as a functionally coupled but physically separate enzyme; in contrast, our findings suggest a direct physical interaction between SAC1 and ORP11 that we expect is necessary in order to deliver lipid or receive from the ORD domain (Nakatsu & Kawasaki, 2021).

**FIGURE 6:**
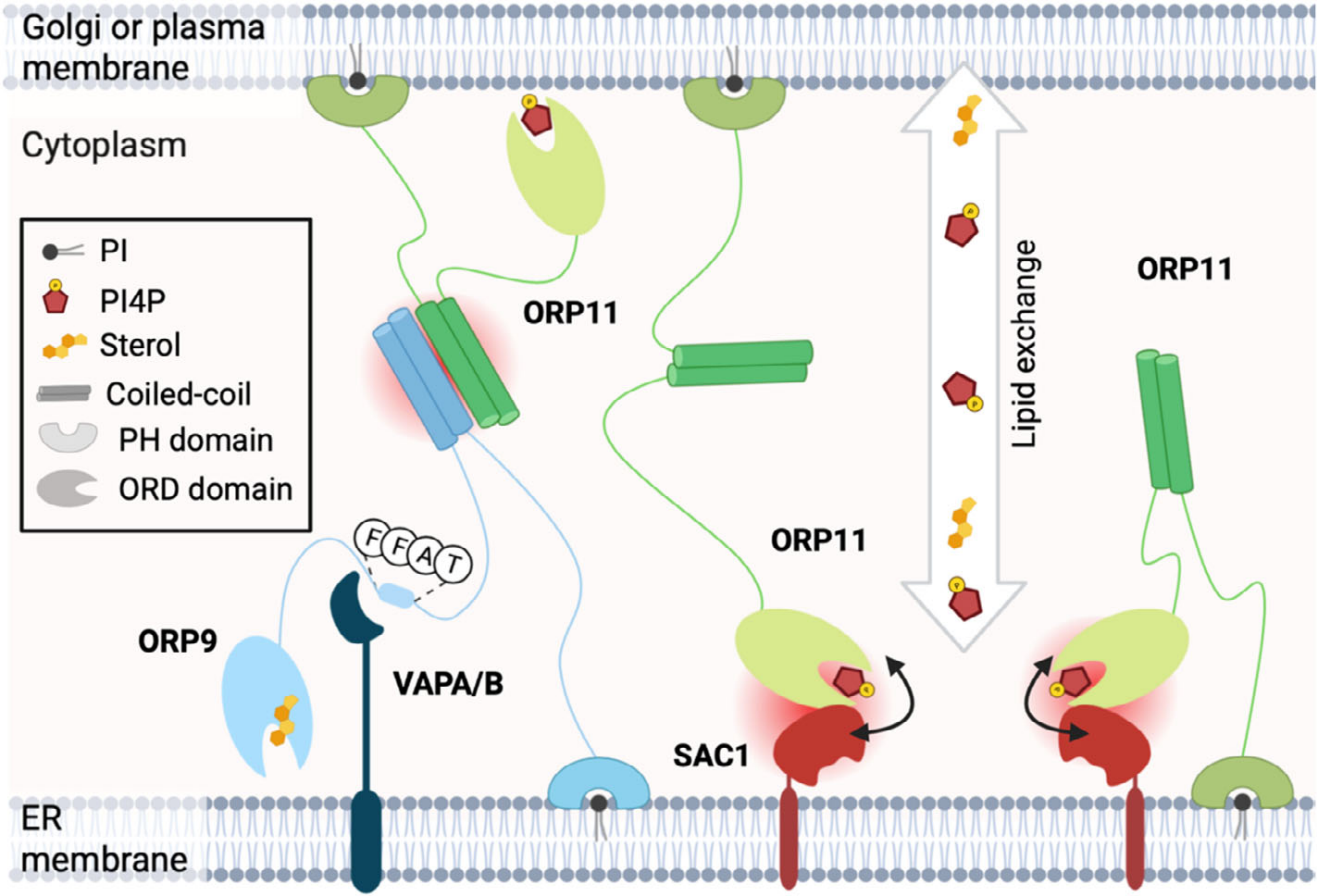
SAC1 recruits a single molecule of ORP11 to the ER. Literature shows that ORPs localize to ER‐PM and ER‐Golgi contact sites. The ORP9‐ORP11 heterodimer, formed through their coiled‐coil domains, bridges these junctions. If bound to VAPA/B via its FFAT motif, ORP9 is anchored to the ER, while ORP11 can occupy the PM membrane surface (shown on the left). If ORP11 is not forming a dimer with ORP9 and acts as a single molecule, we propose that it is recruited by SAC1 to the ER. The ORP11 PH domain could still span the contact site distance and anchor at the PM (shown in the centre), or more simply sit at the ER cytosolic leaflet (shown on the right). SAC1 could: (1) modulate ORP11's lipid transfer activity by dephosphorylating cargo either before export from the ER when it acts as the donor membrane, or after import when it serves as the acceptor membrane; (2) contribute to lipid loading or unloading onto the ORP11 ORD domain spanning the junction. Red circles indicate protein–protein interactions.

Accessory proteins such as SAC1 may play dual roles: facilitating ORP recruitment to ER‐associated MCSs and potentially supporting lipid exchange. SAC1 could act downstream of lipid transfer by converting incoming PI4P to PI, thereby sustaining lipid gradients and directional flow. Strict coupling between lipid transfer and SAC1's enzymatic activity may not be required; mere proximity to the site of PI4P delivery could suffice for efficient exchange. A key question to be investigated is whether ORPs preferentially bind phosphorylated or dephosphorylated lipids, which would clarify their functional interplay. We suggest that ORP11 interacts with SAC1 only as a monomer and not as an ORP9‐ORP11 heterodimer. In this model, SAC1 resides at the ER while the ORP11 ORD domain transiently shuttles between membranes to pick up and deliver lipids. An unresolved issue is whether lipid binding alone is enough to trigger ORD translocation to the opposing membrane, or whether a coordinated mechanism exists in which the PH domain remains bound at the PM or Golgi while the ORD domain engages the ER, alternating positions to shuttle lipids across (Figure 6).

Our results align with the concept that SAC1 activity is tightly regulated by ORP family proteins at MCSs, as previously suggested in yeast and discussed here. In *Saccharomyces cerevisiae*, SAC1 controls PI4P levels across the ER, Golgi, and PM, and its activity requires the oxysterol‐binding homology (Osh) proteins. Notably, Osh3 localizes to ER‐PM contact sites in a PI4P‐dependent manner, and both in vitro and microsomal assays demonstrate that Osh proteins directly stimulate yeast SAC1 phosphatase activity (Stefan et al., 2011). Although attempts to detect a stable complex between the purified yeast SAC1 domain and Osh3 or Osh4 ORDs in vitro were unsuccessful, co‐immunoprecipitation experiments indicated that only a small fraction of SAC1 can interact with Osh proteins in cells (Stefan et al., 2011), consistent with SAC1's approximately 20‐fold higher abundance compared to Osh7 (Ghaemmaghami et al., 2003). These findings suggest a conserved principle whereby ORPs can act both as sensors of membrane lipid composition and as activators of SAC1, enabling localized PI4P turnover and facilitating directional lipid exchange (Knodler et al., 2008; Manford et al., 2010; Stefan et al., 2011).

Prior yeast studies suggested functional coupling between Osh proteins and Sac1, but did not detect ORP‐SAC1 complexes in vitro, in part because experiments used truncated Sac1 constructs and yeast expresses a different set of ORPs and Sac1 paralogs (Stefan et al., 2011). Our structural models provide insight into how human ORPs engage SAC1 at MCSs, offering a mechanistic explanation for interactions that are inherently dynamic: as ORP ORD domains shuttle lipids between membranes, ORP‐SAC1 contacts are short‐lived yet repeatedly formed, allowing efficient lipid transfer and making these transient interactions functionally significant. This also addresses how ORPs lacking canonical FFAT motifs associate with the ER membrane. We propose that SAC1 provides an anchoring role, enabling ORPs to integrate multiple spatial cues. This supports a model of coincidence detection, in which cumulative signals (i.e., membrane localization, lipid recognition, and SAC1 engagement) together drive productive ORP‐SAC1 interactions at ER‐plasma MCSs.

PH domains of ORPs have been reported to act as anchors to the TGN and PM, consistent with the fact that phosphatidylinositol 4‐phosphate (PI4P), their main binding substrate, is largely absent from the ER and concentrated at TGN and PM (He et al., 2023; Kawasaki et al., 2022; Nakatsu & Kawasaki, 2021). This raises the question of how ORPs without FFAT motifs (i.e., ORP11) target the ER. We propose that SAC1 at ER‐MCSs fulfills this role and also acts as a lipid acceptor: PH domains engage PI4P on the TGN or PM, feeding it to the ORP ORD domain for transfer, after which SAC1 dephosphorylates the lipid upon arrival at the ER. This coordinated mechanism links membrane targeting, lipid transfer, and SAC1's enzymatic activity into a single functional cycle.

Overall, all the interactions analyzed in this study may not be strong enough to form stable complexes, but they merit further investigation. In fact, they may operate through coincidence detection, a principle well‐recognized in cell biology. Coincidence detection describes systems in which biological outcomes only arise when multiple conditions are simultaneously satisfied (Mondal et al., 2021; Vicinanza et al., 2019). ORPs and phosphatases co‐residing at MCSs may create the potential for interactions that are weak individually but functionally relevant in the right context. Conversely, the lowest‐confidence predictions, which lack structural alignment with other protein pairs, suggest that direct physical interactions are unlikely or too transient to capture computationally. In these latter cases, interfaces may be too small or weak to model reliably.

### 4.3 Comparative analysis of predicted protein–protein interfaces

We applied an interface lDDT score cutoff of 0.80 for structural alignment using FoldSeek‐Multimer. This threshold was selected to focus on high‐confidence interface similarity after excluding ORP regions not involved in binding. In particular, trimming non‐interface portions of the protein dimers removes residues and incidental contacts from domains such as PH and coiled‐coil regions, thereby enriching for structurally relevant interactions within the ORD domain. Using this cutoff, a coherent subcluster of SAC1‐bound ORPs was identified. This cluster includes both AlphaPulldown2 and AlphaFold3 models, all exhibiting moderate‐to‐high interface confidence metrics (ipTM+pTM >0.50, pDockQ >0.23, actifpTM >0.60) and closely aligned geometries at the SAC1‐ORD interface. Notably, ORP10‐SAC1 and ORP9‐SAC1 display strong structural similarity to ORP11‐SAC1, with consistent alignment across prediction methods (Figure S2).

The FoldSeek‐Multimer methodology is sensitive to broader conformational differences, particularly when large mobile domains like the PH or coiled‐coil regions adopt variable orientations or display disorder. In the case of ORP10‐SAC1, AlphaFold3 and AlphaPulldown both predict similar interface contacts, yet the ORD domain adopts a different angle relative to SAC1 between models. This change likely contributes to a lower interface similarity score. While this could reflect a real difference in predicted complex geometry, several other factors might play a role (Kim et al., 2025). First, FoldSeek‐Multimer aligns structures globally before assessing local interfaces, meaning even small shifts in domain orientation can misalign conserved binding regions. Second, FoldSeek‐Multimer evaluates entire structures chain‐to‐chain and does not align them by selecting interface residues and superimposing them, so well‐conserved binding regions can be underweighted. Third, modest differences in sidechain positioning or flexible loops at the interface which do not affect apparent interface topology can still lower lDDT scores.

In contrast, ORP6‐SAC1, ORP7‐SAC1, ORP5‐SAC1, and ORP8‐SAC1 show consistent binding interfaces across AlphaFold2 and AlphaFold3 models, results that are not seen in the full‐length models being clustered but only when the ORPs were trimmed down to the ORD domain. The predicted contact between SAC1's TM domain and ORP coiled‐coil domains (i.e., in ORP4, 6, 7) is biologically unlikely, reflecting false dimerization due to similar helix packing.

The ORP11‐SAC1 interaction identified here is not reported in existing large‐scale protein–protein interaction (PPI) prediction resources such as PrePPI (Petrey et al., 2023), Predictomes (Schmid & Walter, 2025), or recent AlphaFold‐based human interactome predictions (Zhang et al., 2025). This may reflect methodological differences. These approaches are optimized for proteome‐wide coverage and apply stringent confidence thresholds and classifier‐based filtering to control false positives, which may reduce sensitivity for transient or context‐dependent interactions. In contrast, our protocol adopts a targeted strategy with permissive initial confidence score thresholds from multiple modeling tools (AlphaPulldown2, AlphaFold2‐Multimer, AlphaFold3), combined with analyses such as subcellular localization, membrane insertion, and evolutionary conservation. This highlights the value of context‐aware structural screening for identifying functionally relevant PPIs.

### 4.4 ORP dimerization

Finally, we explored the assembly of higher‐order ORP complexes, specifically focusing on experimentally‐proven ORP9‐ORP11 and potential ORP10‐ORP11 heterodimers. Our attempts to model a similar dimer between ORP10 and ORP11 did not yield confident structural predictions. This interest stems from recent findings showing that ORP9 and ORP11 dimerize to localize efficiently at ER‐Golgi contact sites (Cabukusta et al., 2024). Dimerization occurs through coiled‐coil regions located between the PH and ORD domains (Zhou et al., 2010). Previous analyses using AlphaFold‐Multimer and PCOILS indicate that both ORP9 and ORP11 possess two α‐helices in this region that form a stable dimer through coiled‐coil interactions (Cabukusta et al., 2024). Using a combination of AlphaFold2‐Multimer and AlphaFold3, we successfully modeled two plausible conformations of the experimentally validated ORP9‐ORP11 dimer, including scenarios where SAC1 is simultaneously engaged at the interface.

Our findings loosely align with experimental data: co‐immunoprecipitation experiments demonstrate that ORP11 interacts with VAPA only in the presence of ORP9, and this interaction is abolished in cells lacking ORP9 or expressing a VAPA mutant deficient in FFAT binding (Cabukusta et al., 2024). This confirms that ORP11, although lacking a FFAT motif of its own, can access ER tethers indirectly via dimerization with FFAT‐containing ORPs. The PH domains of both ORP9 and ORP11 mediate Golgi localization through interaction with phosphatidylinositol phosphates (Zhou et al., 2010), anchoring the complex at ER‐Golgi contact sites. Finally, we did not model ORP paralogues forming homodimers, as experimental evidence from co‐immunoprecipitation assays shows that ORP9 and ORP10 do not form homodimers (He et al., 2023; Tan & Finkel, 2022).

Taken together, our results reveal a structurally and biologically coherent model in which SAC1 not only interacts directly with ORP11 but may also serve as a general hub for recruiting FFAT‐deficient ORPs to ER membranes. Given that ORP11 forms heterodimers with ORP9, this raises the intriguing possibility that SAC1, ORP11, and ORP9 form a trimeric complex at MCSs. These interactions exemplify the modularity of the ORP family and underscore the value of high‐confidence in silico predictions guided by proteomic and cellular context. They suggest that MCSs are shaped not just by strong, stoichiometric interactions but by a network of opportunistic, context‐dependent associations, many of which may be mediated by coincidence detection mechanisms. In this light, structure prediction becomes a way to map dynamic interfaces central to organelle communication other than just to identify stable complexes.

## 5 CONCLUSION

Our study demonstrates how machine learning‐based structural prediction can be integrated with proteomic, subcellular localization, and evolutionary data to uncover biologically relevant interactions at MCSs. By applying AlphaPulldown2 and AlphaFold3 at scale, and introducing confidence metrics such as actifpTM, interface lDDT, and FoldSeek‐Multimer clustering, we mapped a structural interaction landscape between lipid transfer proteins and PI phosphatases. Among these, the ORP11‐SAC1 complex stands out as a high‐confidence interaction that satisfies structural, biochemical, and contextual criteria. Our findings also suggest that SAC1 may act not only as a phosphatase but as a recruiter or stabilizer for FFAT‐lacking ORPs at ER‐associated sites. Attempts to refine models with lipid molecules highlight the potential and current limits of applying structure prediction tools to membrane‐bound and ‐aware protein‐lipid systems. Altogether, this work provides a blueprint for how AI‐guided structure prediction, when paired with biological reasoning, can advance our understanding of dynamic, low‐affinity, and context‐dependent interactions that are crucial at organelle contact sites.

## AUTHOR CONTRIBUTIONS

**Filippo Dall'Armellina:** Investigation; writing – original draft; writing – review and editing; formal analysis; data curation; conceptualization; visualization. **Sylvie Urbé:** Conceptualization; methodology; supervision; writing – review and editing. **Daniel J. Rigden:** Conceptualization; methodology; supervision; project administration; resources; writing – review and editing.

## Supporting information


**Figure S1.** (A) AlphaFold3 and AlphaFold2‐Multimer ORP9‐SAC1 complexes superimposed. (B) AlphaFold2‐Multimer ORP9‐SAC1 and ORP11‐SAC1 complexes superimposed (center). PAE heatmaps and interaction confidence scores for the AlphaFold3 and AlphaFold2‐Multimer ORP9‐SAC1 models on the right. “ipTM+pTM” represents the weighted confidence score, calculated as 0.8 × ipTM +0.2 × pTM. “ipSAE” represents the ipSAE_d0dom score.
**Figure S2.** (A) Structural models of the ORP10‐SAC1 and ORP11‐SAC1 complexes generated using AlphaFold3 and AlphaPulldown2, superimposed and visualized in PyMOL. Models are shown with chain‐based coloring to distinguish individual protein components. (B) Same structural models as in (A) but shown with coloring by protein pair object to highlight the interface and interaction regions for each complex. (C) Focused comparison of ORP10‐SAC1 complexes only. AlphaFold3 and AlphaPulldown2 models are shown with domain‐based coloring (left) and whole‐object coloring (center). The corresponding PAE heatmaps for the ORP10‐SAC1 models from AlphaFold3 and AlphaPulldown2 are shown on the right.
**Figure S3.** AlphaFold3 and AlphaPulldown2 models of the ORP11‐SAC1 complex, colored by per‐residue pLDDT confidence scores. Color scheme follows the AlphaFold standard (credit: Konstantin Korotkov).
**Figure S4.** HeliQuest α‐helix amphipathicity calculation. (A) Top‐scoring helical segment. (B) AlphaPulldown2 ORP11‐SAC1 model highlighting regions in SAC1 queried to HeliQuest: 101–137, 424–450, 464–500aa. On the right: helical wheel plot of 119–135aa.
**Figure S5.** (A) AlphaFold3 dimer model of ORP10‐ORP11 colored by chain. On the right, scoring metrics and PAE heatmap. “ipTM+pTM” represents the weighted confidence score, calculated as 0.8 × ipTM +0.2 × pTM. “Interface ipSAE” represents the ipSAE_d0dom score. (B) Same model colored by domain architecture. (C) Colored by per‐residue pLDDT confidence scores. Color scheme follows the AlphaFold standard (credit: Konstantin Korotkov). (D) Colored by per‐residue CoCoNat confidence score for predicting coiled‐coil domains on a scale 0–1.
**Figure S6.** (A) AlphaFold2 dimer model of ORP10‐ORP11 colored by chain. On the right, scoring metrics and PAE heatmap. “ipTM+pTM” represents the weighted confidence score, calculated as 0.8 × ipTM +0.2 × pTM. “Interface ipSAE” represents the ipSAE_d0dom score. (B) Same model colored by domain architecture. (C) Colored by per‐residue pLDDT confidence scores. Color scheme follows the AlphaFold standard (credit: Konstantin Korotkov).
**Figure S7.** (A) AlphaFold3 model of ORP10‐ORP11‐SAC1 colored by chain. On the right, scoring metrics and PAE heatmap. (B) AlphaFold2 model of ORP10‐ORP11‐SAC1 colored by chain. On the right, scoring metrics and PAE heatmap. “ipTM+pTM” represents the weighted confidence score, calculated as 0.8 × ipTM +0.2 × pTM. “Interface ipSAE” represents the ipSAE_d0dom score. (C) AlphaFold2 model of ORP10‐ORP11‐SAC1 colored by per‐residue pLDDT confidence scores. Color scheme follows the AlphaFold standard (credit: Konstantin Korotkov).
**Figure S8.** (A) AlphaFold3 ORP10‐ORP11 dimer modeled with two SAC1 proteins colored by chain (left) and by per‐residue pLDDT confidence scores (right). Color scheme follows the AlphaFold standard (credit: Konstantin Korotkov). On the right, scoring metrics and PAE heatmap. (B) AlphaFold2 ORP10‐ORP11 dimer modeled with two SAC1 proteins colored by chain (left) and by per‐residue pLDDT confidence scores (right). On the right, scoring metrics and PAE heatmap. “ipTM+pTM” represents the weighted confidence score, calculated as 0.8 × ipTM +0.2 × pTM. “Interface ipSAE” represents the ipSAE_d0dom score.
**Figure S9.** (A) AlphaFold3 ORP9‐ORP11 dimer, (B) ORP9‐ORP11 dimer with SAC1, and (C) ORP9‐ORP11 dimer modeled with two SAC1 proteins colored by chain (left) and by per‐residue pLDDT confidence scores (right). Color scheme follows the AlphaFold standard (credit: Konstantin Korotkov). On the right, scoring metrics and PAE heatmap. “ipTM+pTM” represents the weighted confidence score, calculated as 0.8 × ipTM +0.2 × pTM. “Interface ipSAE” represents the ipSAE_d0dom score.
**Figure S10.** (A) AlphaFold2‐Multimer ORP9‐ORP11 dimer, (B) ORP9‐ORP11 dimer with SAC1, and (C) ORP9‐ORP11 dimer modeled with two SAC1 proteins colored by chain (left) and by per‐residue pLDDT confidence scores (right). Color scheme follows the AlphaFold standard (credit: Konstantin Korotkov). On the right, scoring metrics and PAE heatmap. “ipTM+pTM” represents the weighted confidence score, calculated as 0.8 × ipTM +0.2 × pTM. “Interface ipSAE” represents the ipSAE_d0dom score.
**Figure S11.** Subcellular localisation of ORP family proteins and PIPs. (A) Human Cell Map dataset (adapted Go et al., 2021). (B) Map of the Cell cellular compartment annotations in HeLa cells (Itzhak et al., 2016). (C) Prolocate dataset (adapted Jadot et al., 2017), based on organelle fractionation of rat liver. Results are shown for two experimental conditions: A, fasted; B, fed.
**Table S1.** Pairwise sequence identity (%) between human ORP proteins. Sequences were aligned using ProbCons with default parameters in Jalview (Do et al., 2005; Troshin et al., 2011). Values in the table indicate the percentage of identical residues between each pair of sequences. Diagonal entries correspond to self‐comparisons (100% identity).
**Table S2.** Pairwise sequence identity (%) between ORD domains of human ORP proteins. Sequences were aligned using ProbCons with default parameters in Jalview (Do et al., 2005; Troshin et al., 2011). Values in the table indicate the percentage of identical residues between each pair of sequences. Diagonal entries correspond to self‐comparisons (100% identity).
**Table S3.** Lipid composition of the simulated membrane system. Lipid composition of the bilayer generated using CHARMM‐GUI Membrane Builder. The number of lipid molecules in the upper and lower leaflets is reported for each lipid species. The composition was based on the CHARMM‐GUI Archive 18 Biomembranes (Jo et al., 2008; Zhang et al., 2025) (available at https://charmm-gui.org/?doc=archive&lib=biomembrane).
**Table S4.** Membrane system dimensions and area estimates from CHARMM‐GUI. Membrane area estimates and system dimensions calculated by CHARMM‐GUI Membrane Builder. Areas correspond to leaflet‐specific contributions from protein and lipids. Box dimensions refer to the XY dimensions of the simulation cell. Box dimension A (Å) is the box length along X. Box dimension B (Å) is the box length along Y. Together, A × B defines the membrane plane size (XY plane).
**Table S5.** AlphaFold‐driven ORP‐SAC1 interactions in humans. These details can be found on ModelArchive (available at https://modelarchive.org/doi/10.5452/ma‐djr‐af‐orp).
**Table S6.** Proteins mentioned and studied. This table lists all proteins referenced or experimentally analyzed in this work, together with their corresponding UniProt accession numbers. UniProt identifiers were used to unambiguously define protein sequences and annotations and to ensure consistency with publicly curated databases (Tan & Finkel, 2022).

## Data Availability

Raw and processed data generated in this project are publicly accessible. AlphaFold SAC1‐ORP protein models were also reposited on the open access platform Figshare (available at https://doi.org/10.6084/m9.figshare.29769251). An overview of the key models and scores can be accessed via ModelArchive (available at https://modelarchive.org/doi/10.5452/ma-djr-af-orp). Protein names and accession codes used for modeling and bioinformatics analyses are listed in Table S6. Models were reposited in Figshare, while the SAC1‐ORP dimer cluster is available on ModelArchive (Tauriello et al., 2025).
