# Supplementary material for: AlphaFold‐driven discovery of oxysterol‐binding protein‐related protein‐phosphoinositide 3‐, 4‐, and 5‐phosphatase interactions using new generation confidence scores
Source: Protein Sci. 2026 Apr 15;35(5):e70572. doi: 10.1002/pro.70572 (PMC13081691; doi:10.1002/pro.70572)
Supplement: Supplementary file 1 — Figure S1. (A) AlphaFold3 and AlphaFold2‐Multimer ORP9‐SAC1 complexes superimposed. (B) AlphaFold2‐Multimer ORP9‐SAC1 and ORP11‐SAC1 complexes superimposed (center). PAE heatmaps and interaction confidence scores for the AlphaFold3 and AlphaFold2‐Multimer ORP9‐SAC1 models on the right. “ipTM+pTM” represents the weighted confidence score, calculated as 0.8 × ipTM +0.2 × pTM. “ipSAE” represents the ipSAE_d0dom score. Figure S2. (A) Structural models of the ORP10‐SAC1 and ORP11‐SAC1 complexes generated using AlphaFold3 and AlphaPulldown2, superimposed and visualized in PyMOL. Models are shown with chain‐based coloring to distinguish individual protein components. (B) Same structural models as in (A) but shown with coloring by protein pair object to highlight the interface and interaction regions for each complex. (C) Focused comparison of ORP10‐SAC1 complexes only. AlphaFold3 and AlphaPulldown2 models are shown with domain‐based coloring (left) and whole‐object coloring (center). The corresponding PAE heatmaps for the ORP10‐SAC1 models from AlphaFold3 and AlphaPulldown2 are shown on the right. Figure S3. AlphaFold3 and AlphaPulldown2 models of the ORP11‐SAC1 complex, colored by per‐residue pLDDT confidence scores. Color scheme follows the AlphaFold standard (credit: Konstantin Korotkov). Figure S4. HeliQuest α‐helix amphipathicity calculation. (A) Top‐scoring helical segment. (B) AlphaPulldown2 ORP11‐SAC1 model highlighting regions in SAC1 queried to HeliQuest: 101–137, 424–450, 464–500aa. On the right: helical wheel plot of 119–135aa. Figure S5. (A) AlphaFold3 dimer model of ORP10‐ORP11 colored by chain. On the right, scoring metrics and PAE heatmap. “ipTM+pTM” represents the weighted confidence score, calculated as 0.8 × ipTM +0.2 × pTM. “Interface ipSAE” represents the ipSAE_d0dom score. (B) Same model colored by domain architecture. (C) Colored by per‐residue pLDDT confidence scores. Color scheme follows the AlphaFold standard (credit: Konstantin Korotkov). (D) Col [file PRO-35-e70572-s001.docx]

# Supplementary Information

## Figs. S1-S11


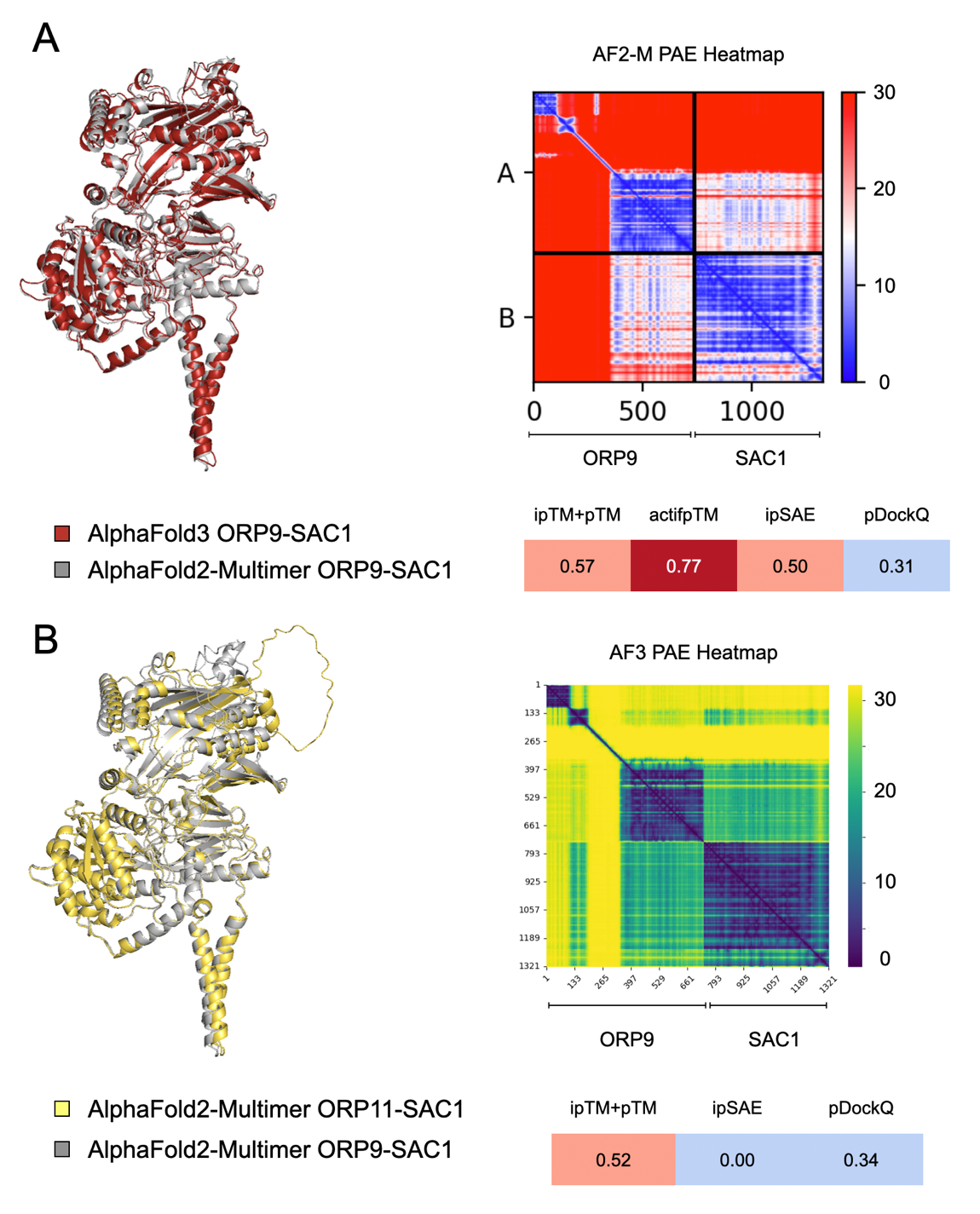


**Fig. S1.** **A.** AlphaFold3 and AlphaFold2-Multimer ORP9-SAC1 complexes superimposed. **B.** AlphaFold2-Multimer ORP9-SAC1 and ORP11-SAC1 complexes superimposed (centre). PAE heatmaps and interaction confidence scores for the AlphaFold3 and AlphaFold2-Multimer ORP9-SAC1 models on the right. "ipTM+pTM" represents the weighted confidence score, calculated as 0.8 × ipTM + 0.2 × pTM. “ipSAE” represents the ipSAE_d0dom score.


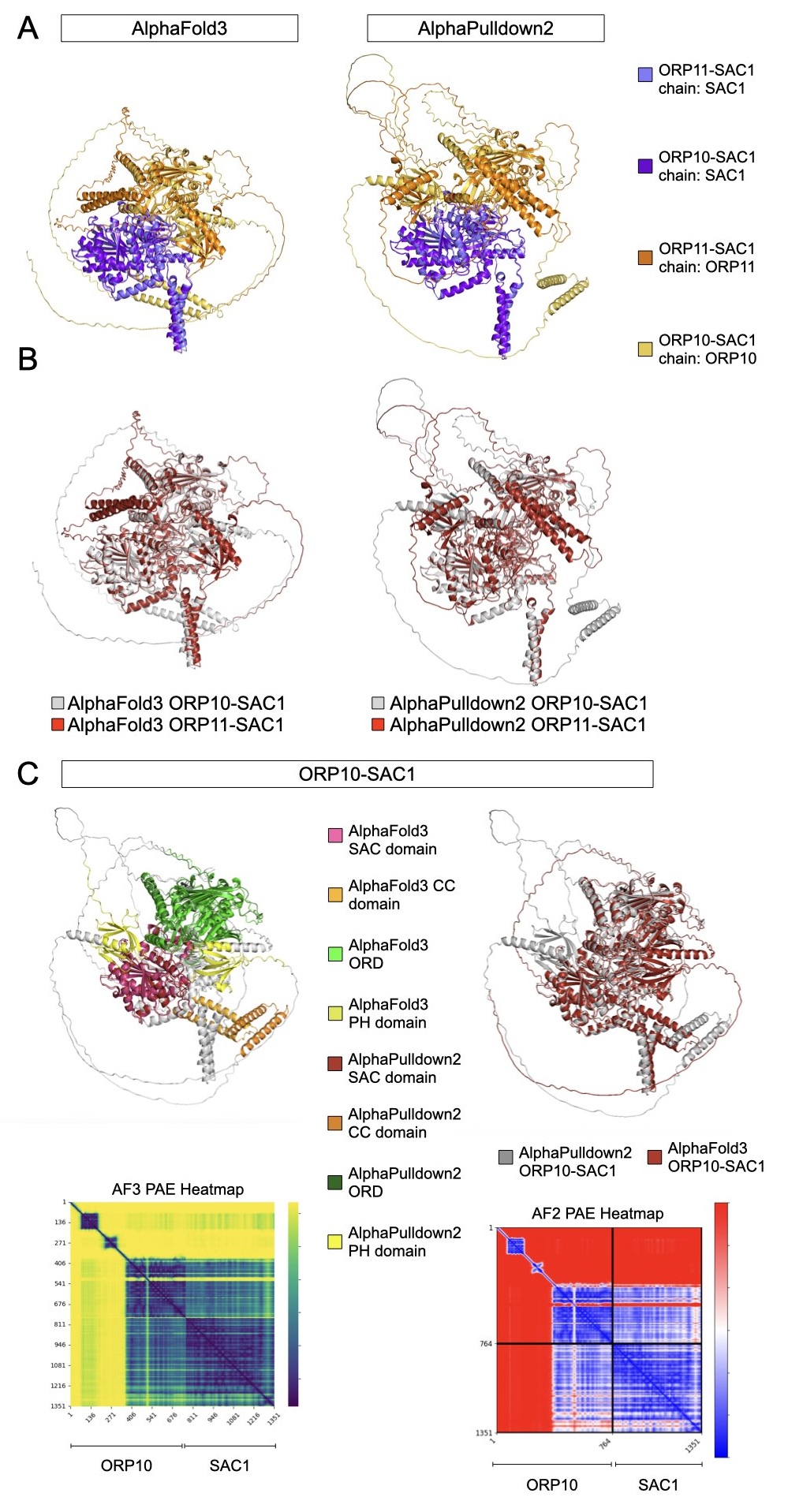


**Fig. S2**. **A.** Structural models of the ORP10-SAC1 and ORP11-SAC1 complexes generated using AlphaFold3 and AlphaPulldown2, superimposed and visualized in PyMOL. Models are shown with chain-based colouring to distinguish individual protein components. **B.** Same structural models as in (A) but shown with colouring by protein pair object to highlight the interface and interaction regions for each complex. **C.** Focused comparison of ORP10-SAC1 complexes only. AlphaFold3 and AlphaPulldown2 models are shown with domain-based colouring (left) and whole-object colouring (centre). The corresponding PAE heatmaps for the ORP10-SAC1 models from AlphaFold3 and AlphaPulldown2 are shown on the right.


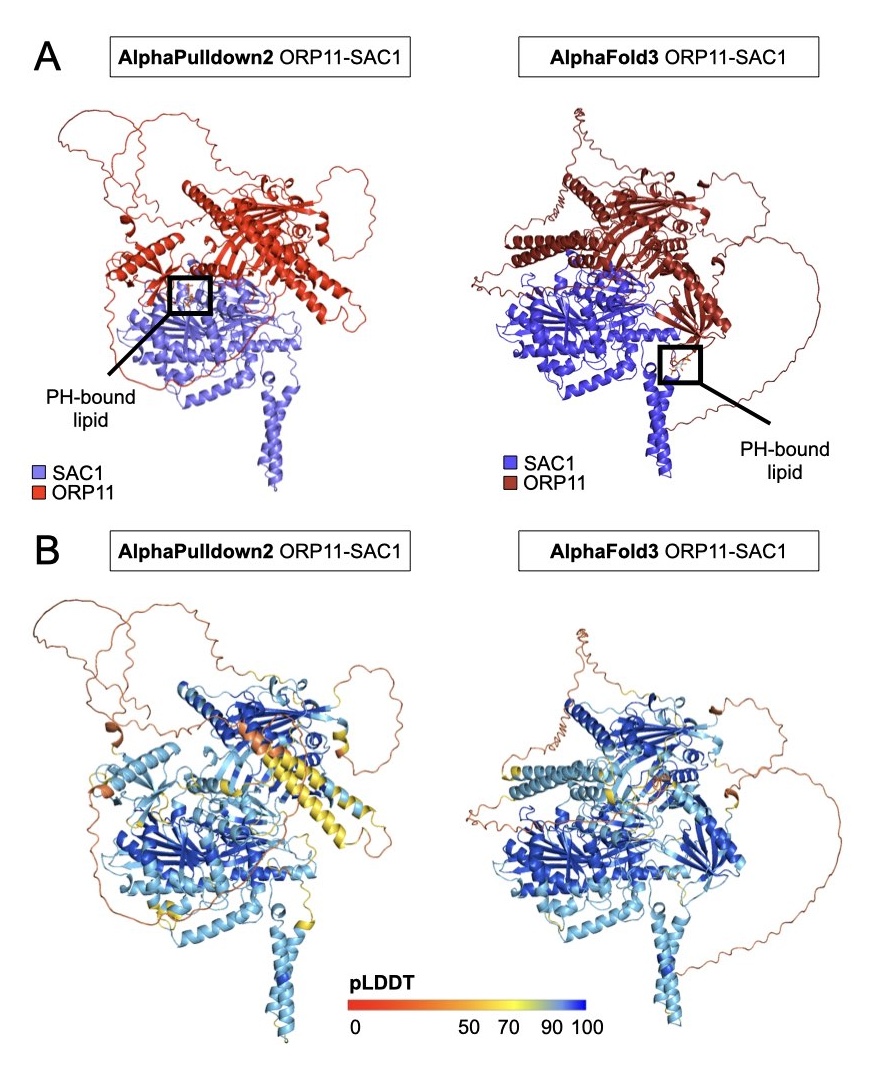


**Fig. S3.** AlphaFold3 and AlphaPulldown2 models of the ORP11-SAC1 complex, coloured by per-residue pLDDT confidence scores. Colour scheme follows the AlphaFold standard (credit: Konstantin Korotkov).


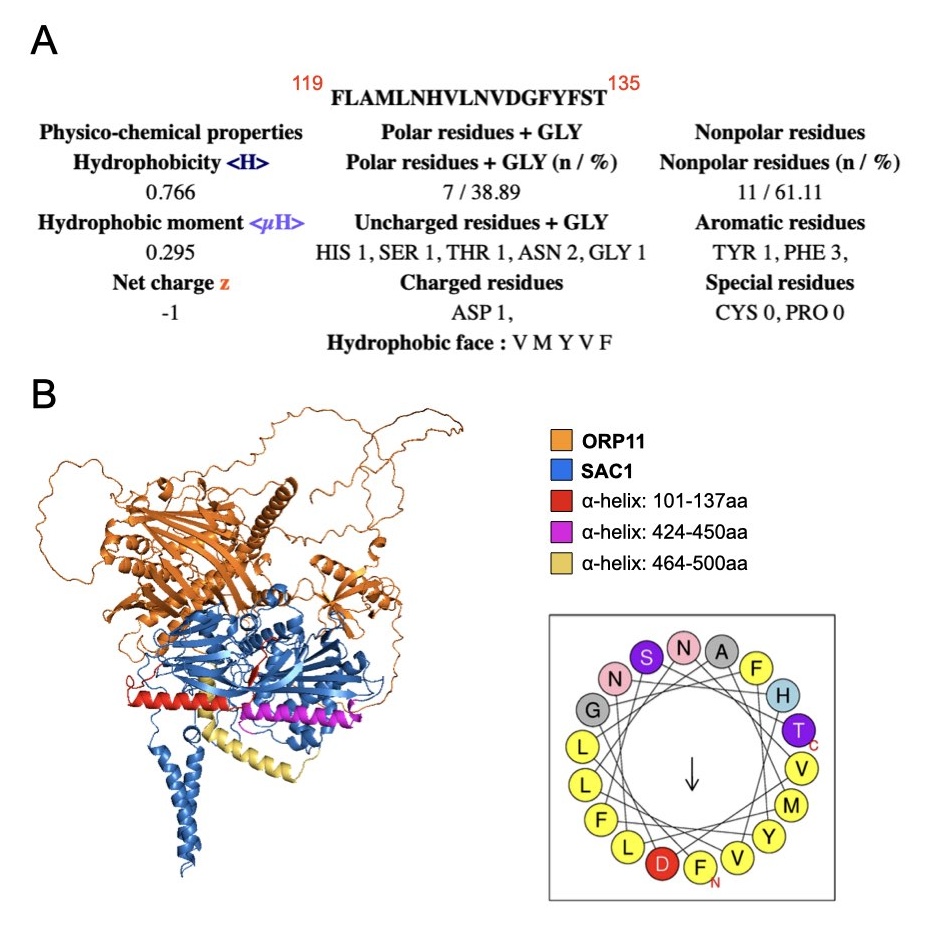


**Fig. S4.** HeliQuest α-helix amphipathicity calculation. **A.** Top-scoring helical segment. **B.** AlphaPulldown2 ORP11-SAC1 model highlighting regions in SAC1 queried to HeliQuest: 101-137, 424-450, 464-500aa. On the right: helical wheel plot of 119-135aa.


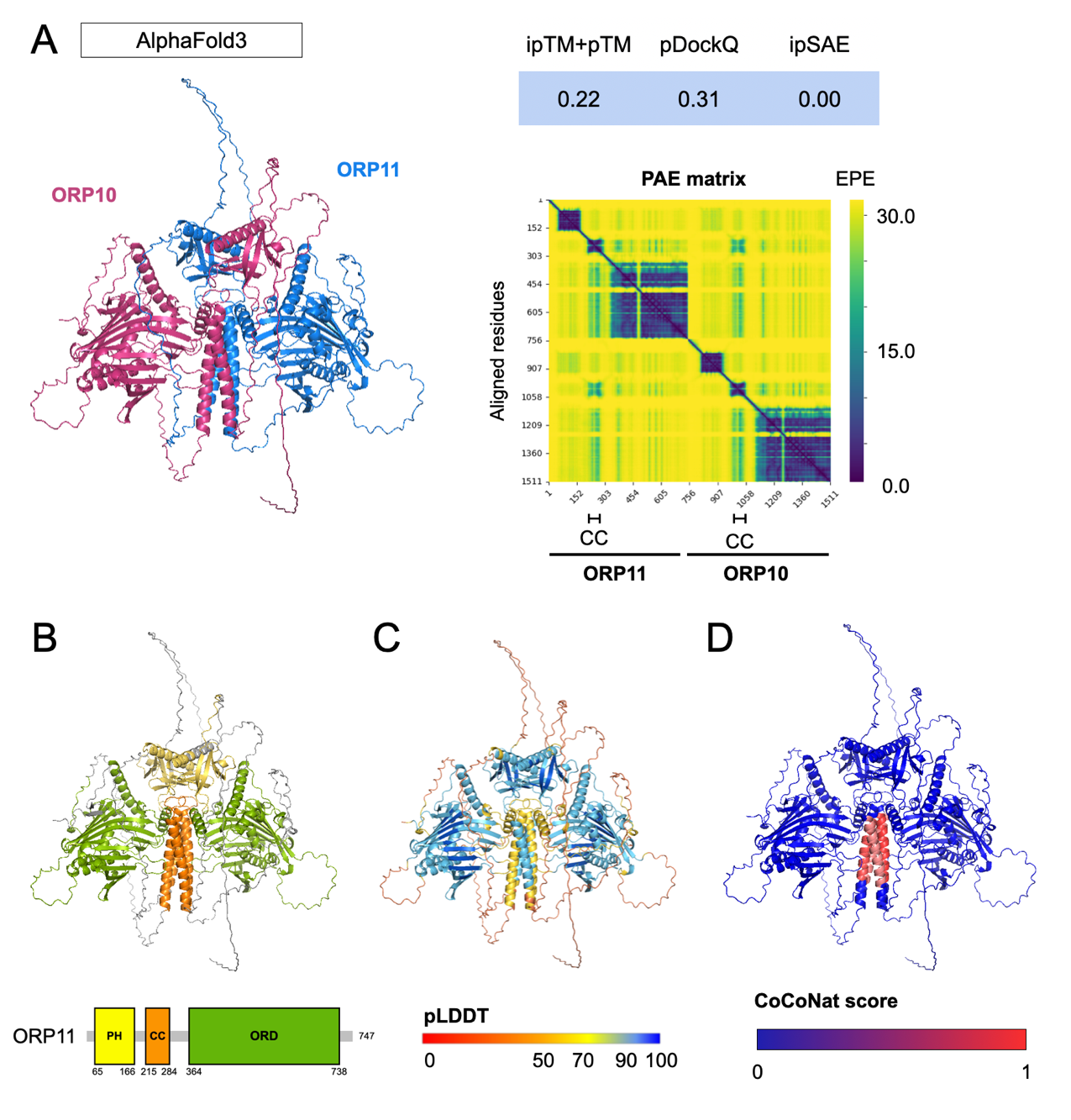


**Fig. S5. A.** AlphaFold3 dimer model of ORP10-ORP11 coloured by chain. On the right, scoring metrics and PAE heatmap. "ipTM+pTM" represents the weighted confidence score, calculated as 0.8 × ipTM + 0.2 × pTM. “Interface ipSAE” represents the ipSAE_d0dom score. **B.** Same model coloured by domain architecture. **C.** Coloured by per-residue pLDDT confidence scores. Colour scheme follows the AlphaFold standard (credit: Konstantin Korotkov). **D.** Coloured by per-residue CoCoNat confidence score for predicting coiled-coil domains on a scale 0-1.


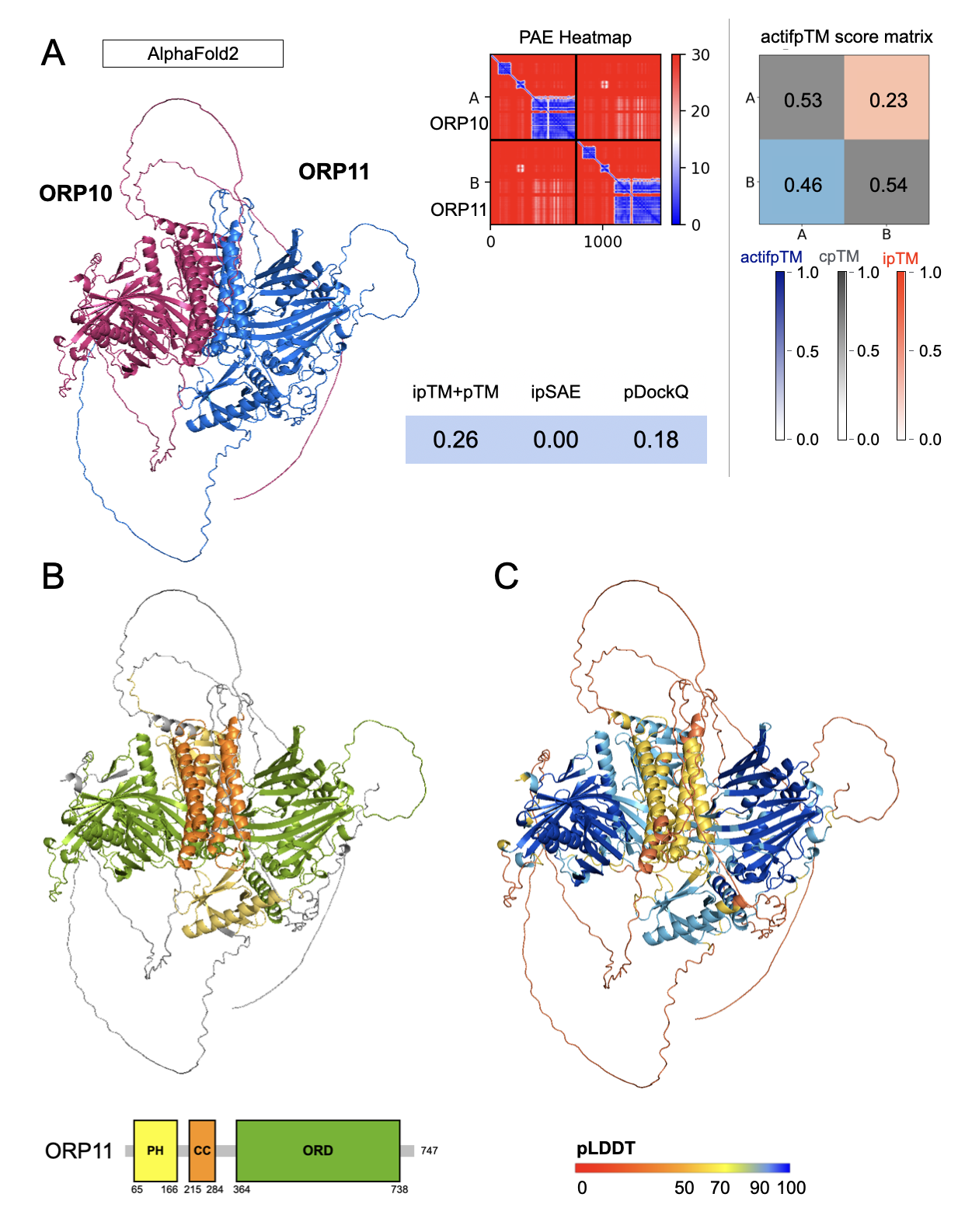


**Fig. S6. A.** AlphaFold2 dimer model of ORP10-ORP11 coloured by chain. On the right, scoring metrics and PAE heatmap. "ipTM+pTM" represents the weighted confidence score, calculated as 0.8 × ipTM + 0.2 × pTM. “Interface ipSAE” represents the ipSAE_d0dom score. **B.** Same model coloured by domain architecture. **C.** Coloured by per-residue pLDDT confidence scores. Colour scheme follows the AlphaFold standard (credit: Konstantin Korotkov).

**
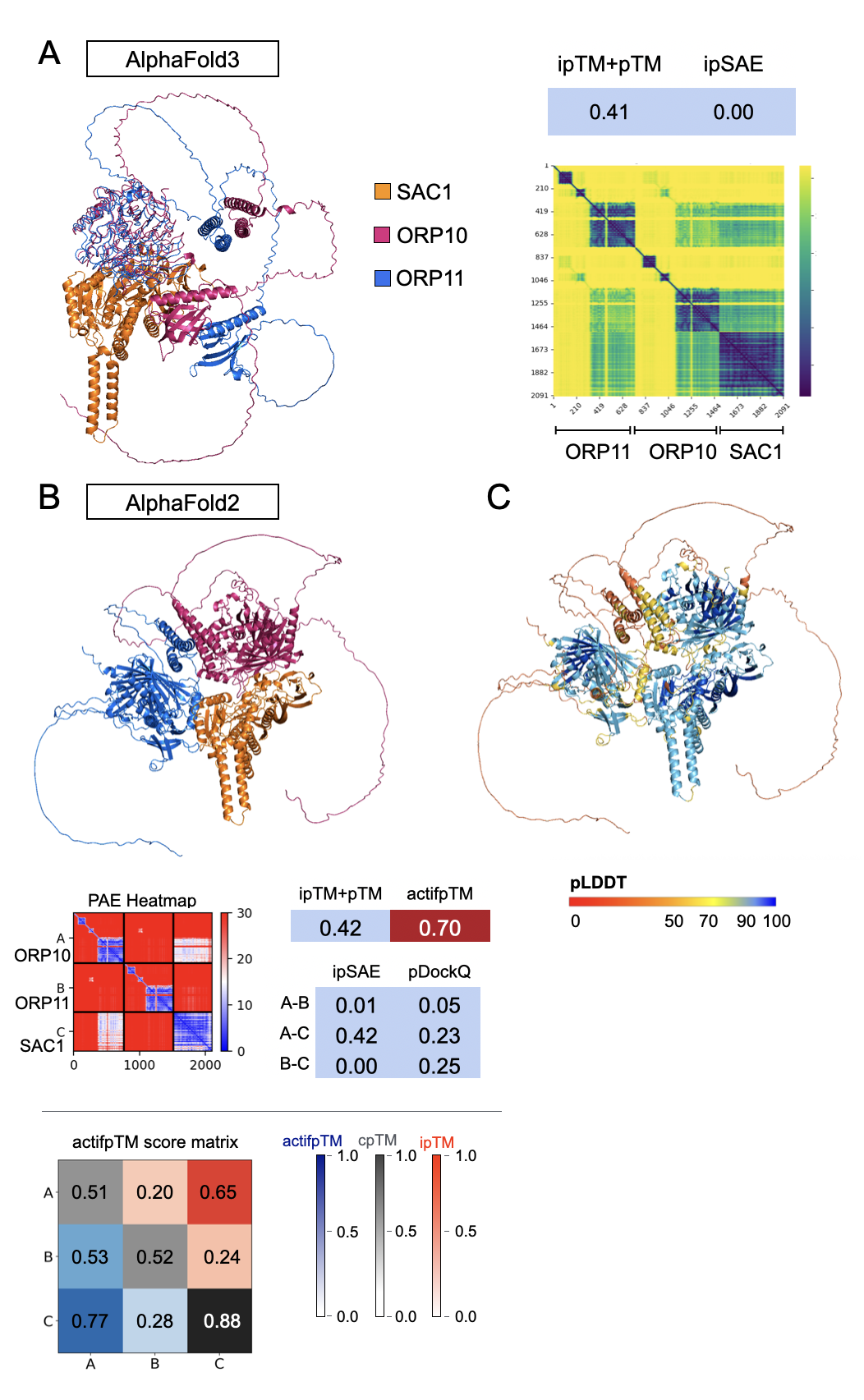
**

**Fig. S7. A.** AlphaFold3 model of ORP10-ORP11-SAC1 coloured by chain. On the right, scoring metrics and PAE heatmap. **B.** AlphaFold2 model of ORP10-ORP11-SAC1 coloured by chain. On the right, scoring metrics and PAE heatmap. "ipTM+pTM" represents the weighted confidence score, calculated as 0.8 × ipTM + 0.2 × pTM. “Interface ipSAE” represents the ipSAE_d0dom score. **C.** AlphaFold2 model of ORP10-ORP11-SAC1 coloured by per-residue pLDDT confidence scores. Colour scheme follows the AlphaFold standard (credit: Konstantin Korotkov).


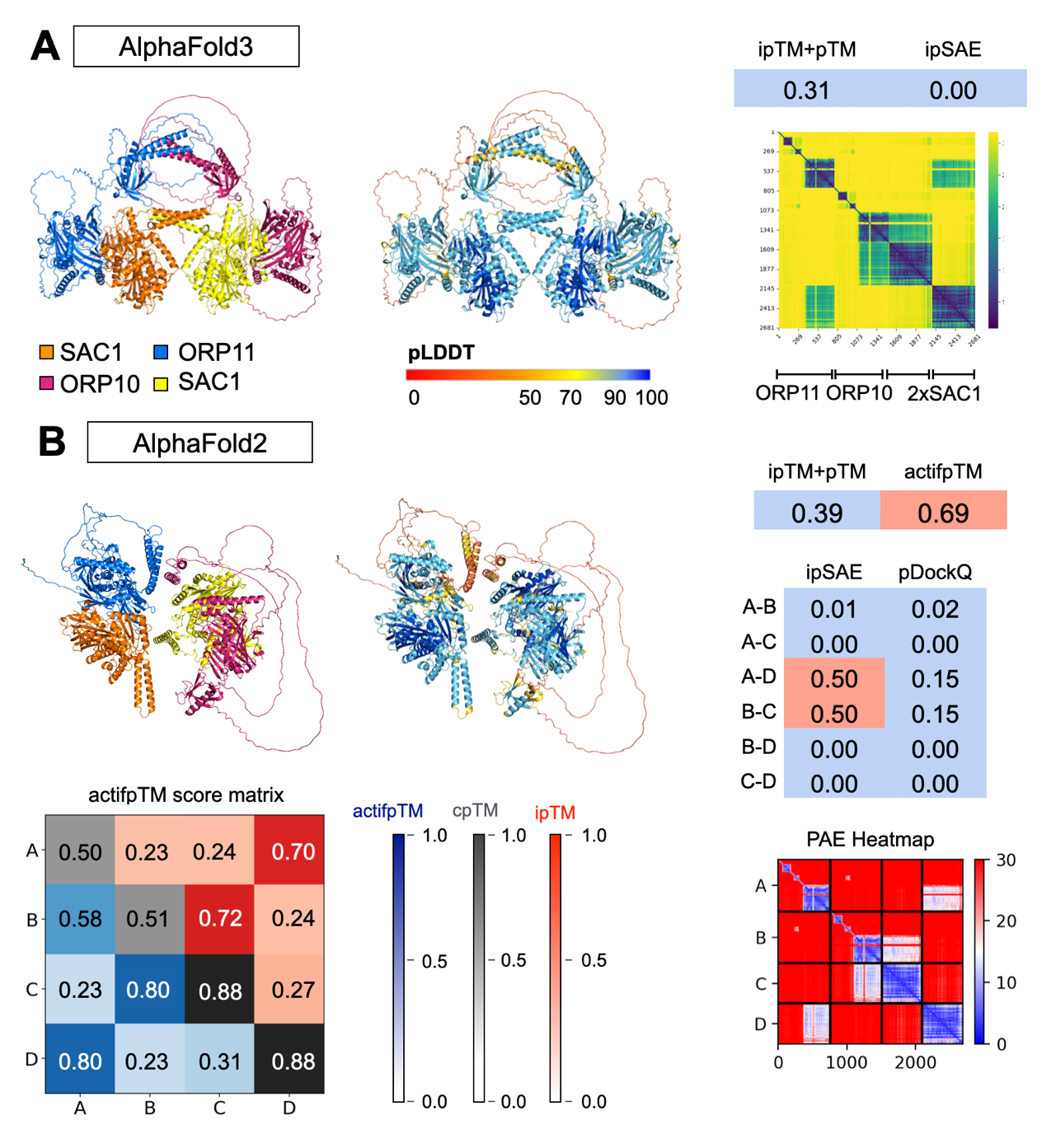


**Fig. S8. A.** AlphaFold3 ORP10-ORP11 dimer modelled with two SAC1 proteins coloured by chain (left) and by per-residue pLDDT confidence scores (right). Colour scheme follows the AlphaFold standard (credit: Konstantin Korotkov). On the right, scoring metrics and PAE heatmap. **B.** AlphaFold2 ORP10-ORP11 dimer modelled with two SAC1 proteins coloured by chain (left) and by per-residue pLDDT confidence scores (right). On the right, scoring metrics and PAE heatmap**.** "ipTM+pTM" represents the weighted confidence score, calculated as 0.8 × ipTM + 0.2 × pTM. “Interface ipSAE” represents the ipSAE_d0dom score.


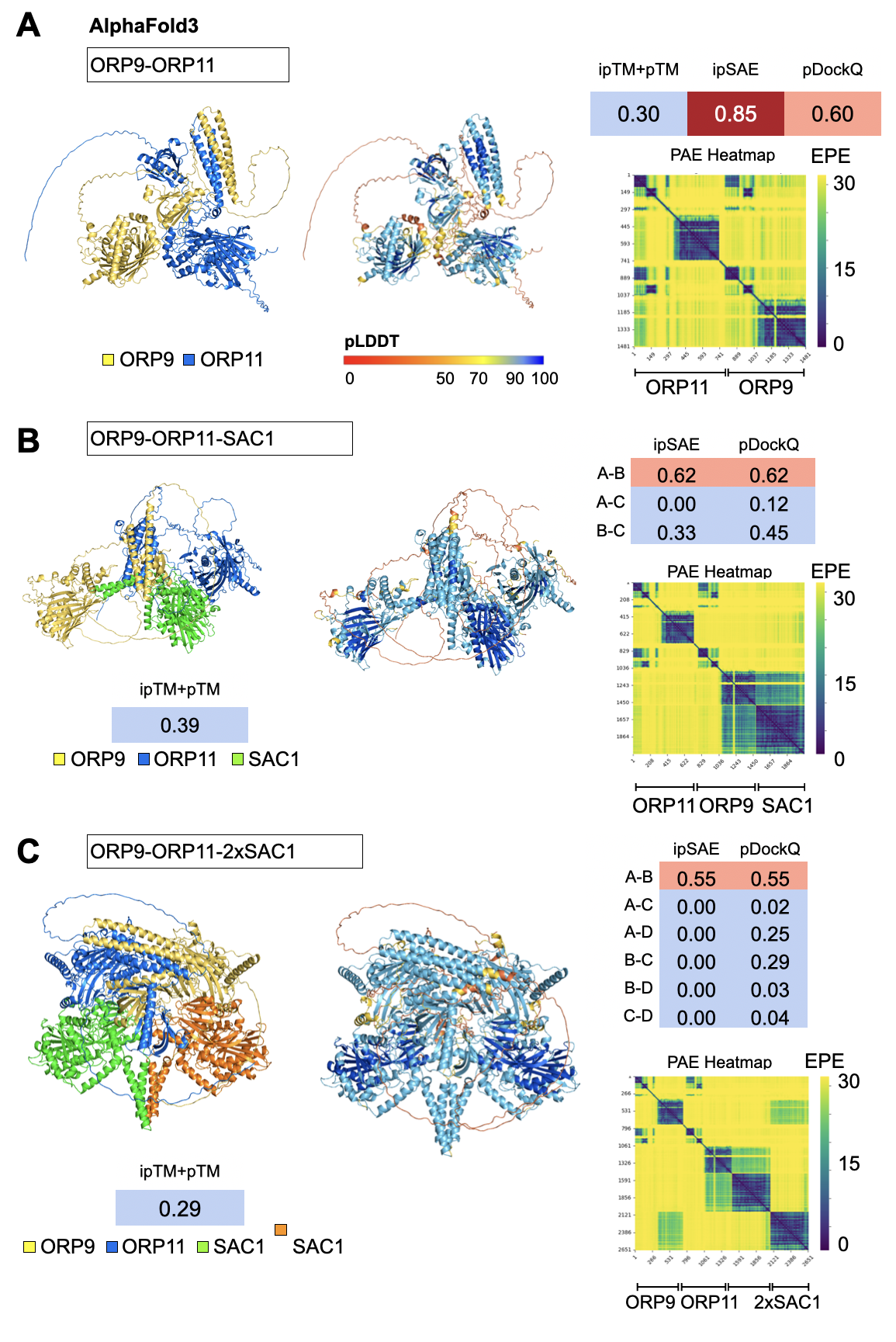


**Fig. S9. A.** AlphaFold3 ORP9-ORP11 dimer, **B.** ORP9-ORP11 dimer with SAC1, and **C.** ORP9-ORP11 dimer modelled with two SAC1 proteins coloured by chain (left) and by per-residue pLDDT confidence scores (right). Colour scheme follows the AlphaFold standard (credit: Konstantin Korotkov). On the right, scoring metrics and PAE heatmap. "ipTM+pTM" represents the weighted confidence score, calculated as 0.8 × ipTM + 0.2 × pTM. “Interface ipSAE” represents the ipSAE_d0dom score.


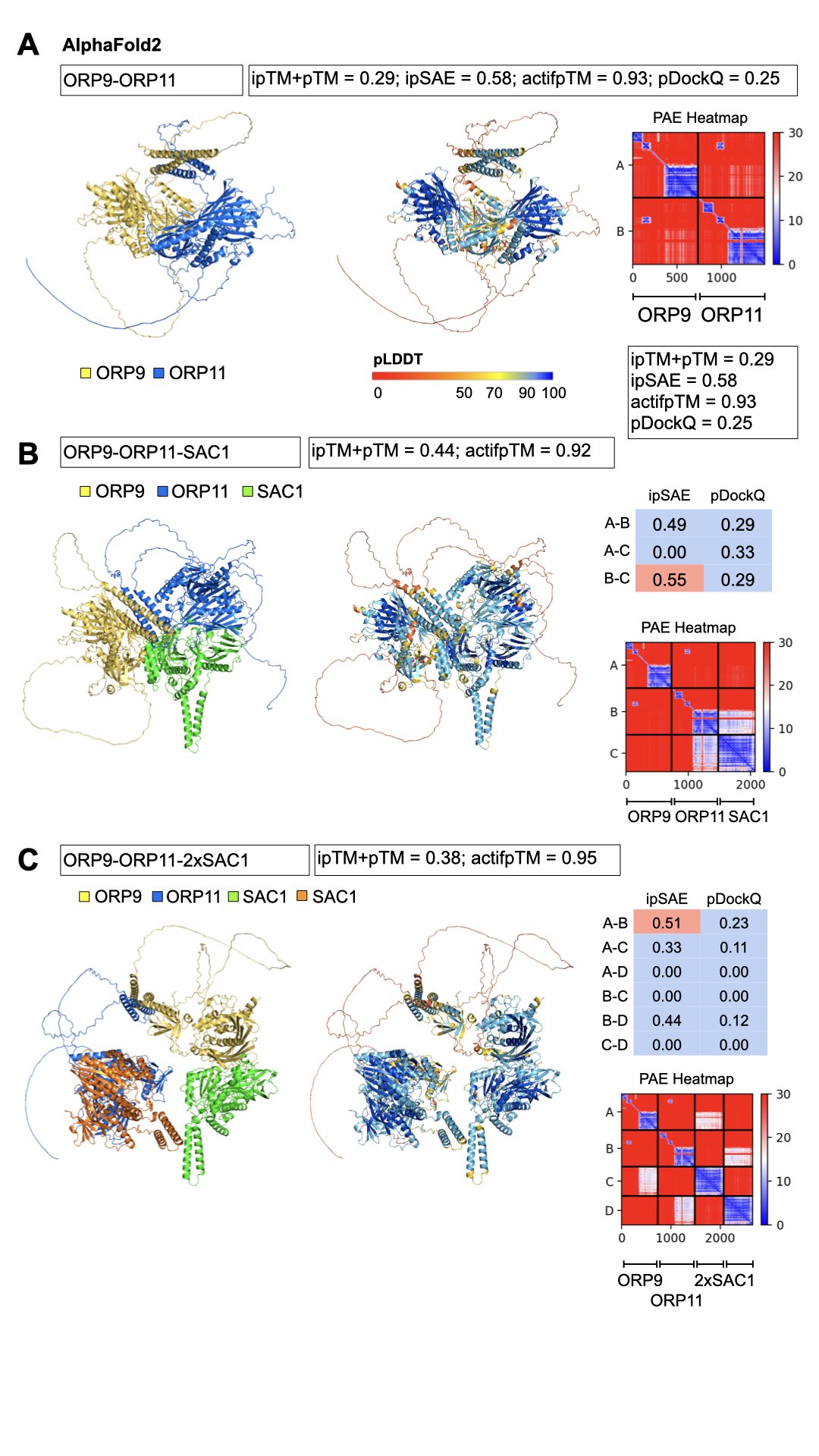


**Fig. S10. A.** AlphaFold2-Multimer ORP9-ORP11 dimer, **B.** ORP9-ORP11 dimer with SAC1, and **C.** ORP9-ORP11 dimer modelled with two SAC1 proteins coloured by chain (left) and by per-residue pLDDT confidence scores (right). Colour scheme follows the AlphaFold standard (credit: Konstantin Korotkov). On the right, scoring metrics and PAE heatmap. "ipTM+pTM" represents the weighted confidence score, calculated as 0.8 × ipTM + 0.2 × pTM. “Interface ipSAE” represents the ipSAE_d0dom score.


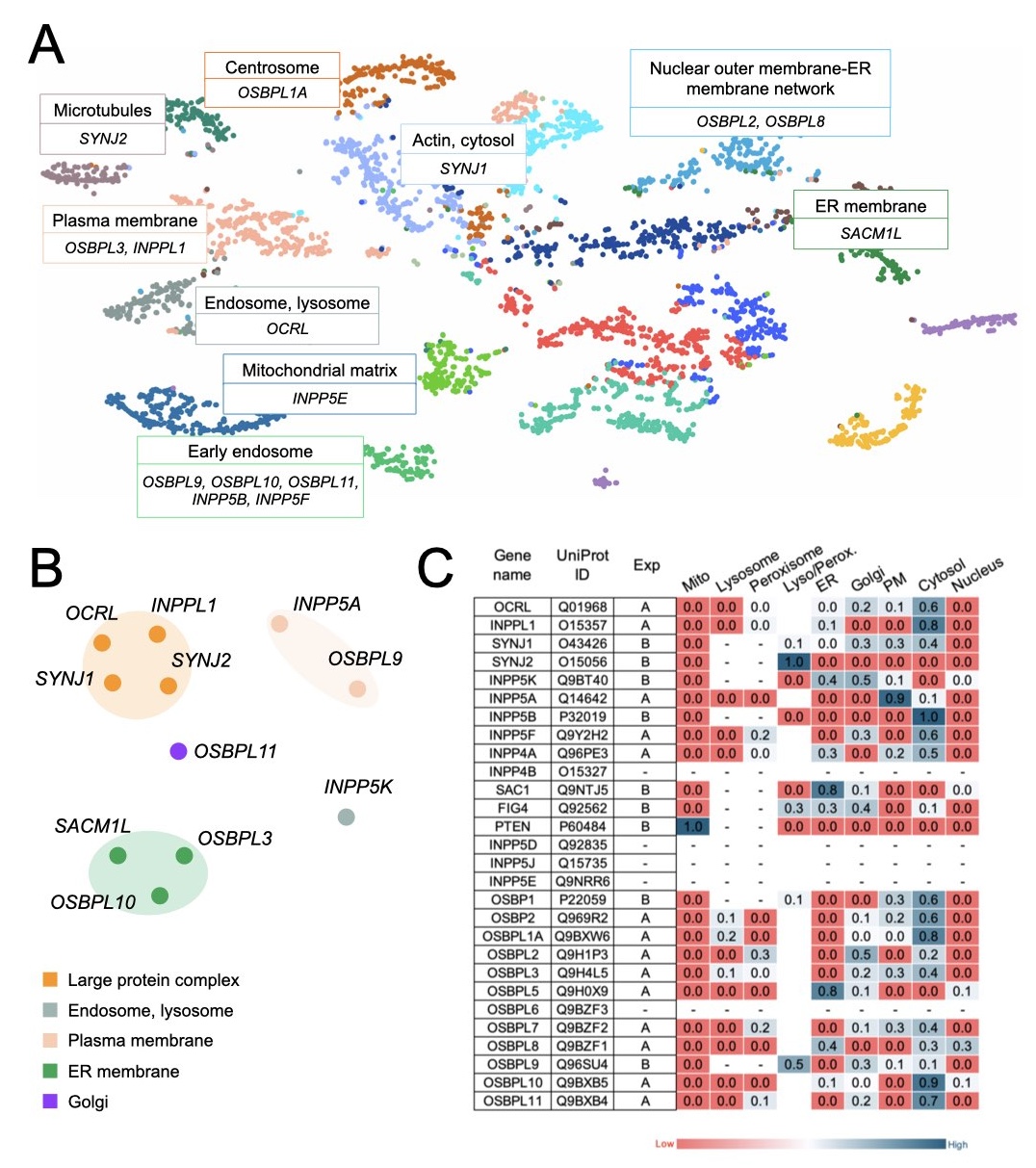


**Fig. S11.** Subcellular localisation of ORP family proteins and PIPs. **A**. Human Cell Map dataset (adapted [^66^](#_ENREF_66)). **B**. Map of the Cell cellular compartment annotations in HeLa cells [^68^](#_ENREF_68). **C**. Prolocate dataset (adapted [^67^](#_ENREF_67)), based on organelle fractionation of rat liver. Results are shown for two experimental conditions: A, fasted; B, fed.

## Tables

**Table S1 Pairwise sequence identity (%) between human ORP proteins.** Sequences were aligned using ProbCons with default parameters in Jalview [^53^](#_ENREF_53)^,^[^54^](#_ENREF_54). Values in the table indicate the percentage of identical residues between each pair of sequences. Diagonal entries correspond to self-comparisons (100% identity).

|  | OSBP | ORP1 | ORP2 | ORP3 | ORP4 | ORP5 | ORP6 | ORP7 | ORP8 | ORP9 | ORP10 | ORP11 |
| --- | --- | --- | --- | --- | --- | --- | --- | --- | --- | --- | --- | --- |
| OSBP | 100 | 30.68 | 37.90 | 27.50 | 58.90 | 20.68 | 27.01 | 27.77 | 20.52 | 22.62 | 20.72 | 21.23 |
| ORP1 |  | 100 | 65.77 | 25.77 | 30.44 | 23.08 | 26.55 | 26.59 | 22.59 | 23.88 | 19.74 | 18.67 |
| ORP2 |  |  | 100 | 37.30 | 35.80 | 26.32 | 36.16 | 34.90 | 26.49 | 23.47 | 20.99 | 20.53 |
| ORP3 |  |  |  | 100 | 26.15 | 20.86 | 57.54 | 49.55 | 20.33 | 19.10 | 19.01 | 17.55 |
| ORP4 |  |  |  |  | 100 | 22.34 | 26.84 | 26.89 | 20.07 | 23.12 | 21.06 | 20.43 |
| ORP5 |  |  |  |  |  | 100 | 20.95 | 21.61 | 54.07 | 25.59 | 26.75 | 25.13 |
| ORP6 |  |  |  |  |  |  | 100 | 49.78 | 19.47 | 19.66 | 18.17 | 18.68 |
| ORP7 |  |  |  |  |  |  |  | 100 | 19.57 | 21.24 | 21.45 | 17.98 |
| ORP8 |  |  |  |  |  |  |  |  | 100 | 27.02 | 25.35 | 24.55 |
| ORP9 |  |  |  |  |  |  |  |  |  | 100 | 37.25 | 39.63 |
| ORP10 |  |  |  |  |  |  |  |  |  |  | 100 | 54.38 |
| ORP11 |  |  |  |  |  |  |  |  |  |  |  | 100 |

**Table S2 Pairwise sequence identity (%) between ORD domains of human ORP proteins.** Sequences were aligned using ProbCons with default parameters in Jalview [^53^](#_ENREF_53)^,^[^54^](#_ENREF_54). Values in the table indicate the percentage of identical residues between each pair of sequences. Diagonal entries correspond to self-comparisons (100% identity).

|  | OSBP | ORP1 | ORP2 | ORP3 | ORP4 | ORP5 | ORP6 | ORP7 | ORP8 | ORP9 | ORP10 | ORP11 |
| --- | --- | --- | --- | --- | --- | --- | --- | --- | --- | --- | --- | --- |
| OSBP | 100 | 42.51 | 40.49 | 41.41 | 67.89 | 25.41 | 40.10 | 39.06 | 25.76 | 22.98 | 21.41 | 20.77 |
| ORP1 |  | 100 | 71.18 | 39.04 | 41.36 | 28.97 | 39.29 | 38.79 | 29.85 | 29.39 | 27.06 | 22.90 |
| ORP2 |  |  | 100 | 39.00 | 39.37 | 26.63 | 38.75 | 37.25 | 27.59 | 28.57 | 23.43 | 22.98 |
| ORP3 |  |  |  | 100 | 38.81 | 25.50 | 70.37 | 70.09 | 23.63 | 25.20 | 21.71 | 20.32 |
| ORP4 |  |  |  |  | 100 | 25.89 | 40.51 | 34.96 | 25.21 | 21.14 | 19.80 | 19.94 |
| ORP5 |  |  |  |  |  | 100 | 26.70 | 24.08 | 69.35 | 38.06 | 34.09 | 35.35 |
| ORP6 |  |  |  |  |  |  | 100 | 68.38 | 26.18 | 24.39 | 22.77 | 20.92 |
| ORP7 |  |  |  |  |  |  |  | 100 | 25.66 | 25.20 | 23.28 | 22.26 |
| ORP8 |  |  |  |  |  |  |  |  | 100 | 41.94 | 36.77 | 34.07 |
| ORP9 |  |  |  |  |  |  |  |  |  | 100 | 57.31 | 56.37 |
| ORP10 |  |  |  |  |  |  |  |  |  |  | 100 | 71.10 |
| ORP11 |  |  |  |  |  |  |  |  |  |  |  | 100 |

**Table S3 Lipid composition of the simulated membrane system**. Lipid composition of the bilayer generated using CHARMM-GUI Membrane Builder. The number of lipid molecules in the upper and lower leaflets is reported for each lipid species. The composition was based on the CHARMM-GUI Archive 18 Biomembranes [^61^](#_ENREF_61)^,^[^81^](#_ENREF_81) (available at https://charmm-gui.org/?doc=archive&lib=biomembrane).

| **Lipid type** | **Upper leaflet (n)** | **Lower leaflet (n)** |
| --- | --- | --- |
| Cholesterol | 20 | 20 |
| POPA | 4 | 4 |
| POPC | 64 | 64 |
| PLPC | 72 | 72 |
| PSPE | 32 | 32 |
| POPE | 20 | 20 |
| OLPS | 12 | 12 |
| SLPI | 12 | 12 |
| SAPI | 12 | 12 |
| SAPC | 108 | 108 |
| SAPE | 28 | 28 |
| PSM | 16 | 16 |
| **Total lipids** | **400** | **400** |

**Table S4 Membrane system dimensions and area estimates from CHARMM-GUI.** Membrane area estimates and system dimensions calculated by CHARMM-GUI Membrane Builder. Areas correspond to leaflet-specific contributions from protein and lipids. Box dimensions refer to the XY dimensions of the simulation cell. Box dimension A (Å) is the box length along X. Box dimension B (Å) is the box length along Y. Together, A × B defines the membrane plane size (XY plane).

| **Parameter** | **Upper leaflet** | **Lower leaflet** |
| --- | --- | --- |
| Protein area (Å²) | 293.19 | 1352.78 |
| Lipid area (Å²) | 25826.4 | 25826.4 |
| Number of lipids | 400 | 400 |
| Total area (Å²) | 26119.59 | 27179.18 |
| **System-wide parameters** | **Value** | |
| Protein X extent (Å) | 57.96 | |
| Protein Y extent (Å) | 60.33 | |
| Average membrane area (Å²) | 26649.39 | |
| Box dimension A (Å) | 163.25 | |
| Box dimension B (Å) | 163.25 | |

Table S5. AlphaFold-driven ORP-SAC1 interactions in humans. These details can be found on ModelArchive (available at <https://modelarchive.org/doi/10.5452/ma-djr-af-orp>).

| **Model** | **Tool** | **ipTM** | **0.8*ipTM+0.2*pTM** | **actifpTM** | **ipSAE** | **pDockQ** |
| --- | --- | --- | --- | --- | --- | --- |
| ORP1-SAC1 | AlphaFold2 | 0.27 | 0.31 | 0.43 | 0.00 | 0.10 |
| ORP2-SAC1 | AlphaFold2 | 0.26 | 0.33 | 0.46 | 0.00 | 0.10 |
| ORP3-SAC1 | AlphaFold2 | 0.40 | 0.42 | 0.55 | 0.01 | 0.10 |
| ORP4-SAC1 | AlphaFold2 | 0.39 | 0.42 | 0.52 | 0.01 | 0.08 |
| ORP5-SAC1 | AlphaFold2 | 0.65 | 0.64 | 0.82 | 0.55 | 0.27 |
| ORP6-SAC1 | AlphaFold2 | 0.75 | 0.72 | 0.87 | 0.69 | 0.37 |
| ORP7-SAC1 | AlphaFold2 | 0.77 | 0.74 | 0.89 | 0.71 | 0.25 |
| ORP8-SAC1 | AlphaFold2 | 0.70 | 0.68 | 0.86 | 0.60 | 0.26 |
| ORP9-SAC1 | AlphaFold2 | 0.56 | 0.57 | 0.77 | 0.50 | 0.31 |
| ORP10-SAC1 | AlphaFold2 | 0.74 | 0.72 | 0.91 | 0.68 | 0.38 |
| ORP11-SAC1 | AlphaFold2 | 0.78 | 0.76 | 0.92 | 0.73 | 0.39 |
| OSBP-SAC1 | AlphaFold2 | 0.30 | 0.34 | 0.45 | 0.00 | 0.11 |
| ORP1-SAC1 | AlphaPulldown2 | 0.30 | 0.34 | N/A | N/A | N/A |
| ORP2-SAC1 | AlphaPulldown2 | 0.27 | 0.34 | N/A | N/A | N/A |
| ORP3-SAC1 | AlphaPulldown2 | 0.52 | 0.53 | N/A | N/A | N/A |
| ORP4-SAC1 | AlphaPulldown2 | 0.53 | 0.54 | N/A | N/A | N/A |
| ORP5-SAC1 | AlphaPulldown2 | 0.51 | 0.52 | N/A | N/A | N/A |
| ORP6-SAC1 | AlphaPulldown2 | 0.47 | 0.48 | N/A | N/A | N/A |
| ORP7-SAC1 | AlphaPulldown2 | 0.63 | 0.62 | N/A | N/A | N/A |
| ORP8-SAC1 | AlphaPulldown2 | 0.49 | 0.51 | N/A | N/A | N/A |
| ORP9-SAC1 | AlphaPulldown2 | 0.52 | 0.53 | N/A | N/A | N/A |
| ORP10-SAC1 | AlphaPulldown2 | 0.79 | 0.76 | N/A | N/A | N/A |
| ORP11-SAC1 | AlphaPulldown2 | 0.81 | 0.79 | N/A | N/A | N/A |
| OSBP-SAC1 | AlphaPulldown2 | 0.54 | 0.55 | N/A | N/A | N/A |
| ORP1-SAC1 | AlphaFold3 | 0.19 | 0.24 | N/A | 0.00 | 0.26 |
| ORP2-SAC1 | AlphaFold3 | 0.14 | 0.22 | N/A | 0.00 | 0.06 |
| ORP3-SAC1 | AlphaFold3 | 0.20 | 0.28 | N/A | 0.00 | 0.37 |
| ORP4-SAC1 | AlphaFold3 | 0.74 | 0.69 | N/A | 0.63 | 0.38 |
| ORP5-SAC1 | AlphaFold3 | 0.21 | 0.26 | N/A | 0.00 | 0.33 |
| ORP6-SAC1 | AlphaFold3 | 0.39 | 0.41 | N/A | 0.00 | 0.33 |
| ORP7-SAC1 | AlphaFold3 | 0.22 | 0.27 | N/A | 0.00 | 0.26 |
| ORP8-SAC1 | AlphaFold3 | 0.29 | 0.33 | N/A | 0.00 | 0.16 |
| ORP9-SAC1 | AlphaFold3 | 0.42 | 0.45 | N/A | 0.00 | 0.35 |
| ORP10-SAC1 | AlphaFold3 | 0.73 | 0.71 | N/A | 0.66 | 0.52 |
| ORP11-SAC1 | AlphaFold3 | 0.71 | 0.70 | N/A | 0.63 | 0.43 |
| OSBP-SAC1 | AlphaFold3 | 0.75 | 0.73 | N/A | 0.68 | 0.34 |

**Table S6. Proteins mentioned and studied.** This table lists all proteins referenced or experimentally analysed in this work, together with their corresponding UniProt accession numbers. UniProt identifiers were used to unambiguously define protein sequences and annotations and to ensure consistency with publicly curated databases [^82^](#_ENREF_82).

| **Protein name** | **UniProt ID** |
| --- | --- |
| INPP5A | Q14642 |
| OCRL | Q01968 |
| INPP5B | P32019 |
| INPP5E | Q9NRR6 |
| INPP5D | Q92835 |
| INPPL1 | O15357 |
| INPP5K | Q9BT40 |
| INPP5J | Q15735 |
| SYNJ1 | O43426 |
| SYNJ2 | O15056 |
| PTEN | P60484 |
| FIG4 | Q92562 |
| SAC1 | Q9NTJ5 |
| INPP4A | Q96PE3 |
| INPP4B | O15327 |
| OSBP | P22059 |
| ORP1 | Q9BXW6 |
| ORP2 | Q9H1P3 |
| ORP3 | Q9H4L5 |
| ORP4 | Q969R2 |
| ORP5 | Q9H0X9 |
| ORP6 | Q9BZF3 |
| ORP7 | Q9BZF2 |
| ORP8 | Q9BZF1 |
| ORP9 | Q96SU4 |
| ORP10 | Q9BXB5 |
| ORP11 | Q9BXB4 |
